# A Reassessment of the Relationship between GDP and Life Satisfaction

**DOI:** 10.1371/journal.pone.0079358

**Published:** 2013-11-27

**Authors:** Eugenio Proto, Aldo Rustichini

**Affiliations:** 1 Department of Economics and CAGE, University of Warwick, Coventry, United Kingdom; 2 Department of Economics, University of Minnesota, Minneapolis, Minnesota, United States; University of Buenos Aires, Argentina

## Abstract

The scientific debate on the relation between Gross Domestic Product (GDP) and self reported indices of life satisfaction is still open. In a well-known finding, Easterlin reported no significant relationship between happiness and aggregate income in time-series analysis. However, life satisfaction appears to be strictly monotonically increasing with income when one studies this relation at a point in time across nations. Here, we analyze the relation between per capita GDP and life satisfaction without imposing a functional form and eliminating potentially confounding country-specific factors. We show that this relation clearly increases in country with a per capita GDP below 15,000 USD (2005 in Purchasing Power Parity), then it flattens for richer countries. The probability of reporting the highest level of life satisfaction is more than 12% lower in the poor countries with a per capita GDP below 5,600 USD than in the counties with a per capita GDP of about 15,000 USD. In countries with an income above 17,000 USD the probability of reporting the highest level of life satisfaction changes within a range of 2% maximum. Interestingly enough, life satisfaction seems to peak at around 30,000 USD and then slightly but significantly decline among the richest countries. These results suggest an explanation of the Easterlin paradox: life satisfaction increases with GDP in poor country, but this relation is approximately flat in richer countries. We explain this relation with aspiration levels. We assume that a gap between aspiration and realized income is negatively perceived; and aspirations to higher income increase with income. These facts together have a negative effect on life satisfaction, opposite to the positive direct effect of the income. The net effect is ambiguous. We predict a higher negative effect in individuals with higher sensitivity to losses (measured by their neuroticism score) and provide econometric support of this explanation.

## Introduction

The debate on whether higher income in a country is associated with higher life satisfaction is considered of crucial importance for scientific and for policy reasons. For example, if one thinks that the answer to the question is fundamentally affirmative, then alternative measurements of the wealth of a nation are redundant, and traditional values gross domestic product measures suffice. Instead, if the answer is negative, then there is a fundamental need to re-evaluate what public policies take as criterion of performance.

The debate is still open. In a well-known finding, [1] reported no significant relationship between happiness and aggregate income in time-series analysis. For example, Easterlin shows that the income per capita in the USA in the period 1974–2004 almost doubled, but the average level of happiness showed no appreciable trend upwards. This puzzling finding, appropriately called the Easterlin Paradox, has been confirmed in similar studies by psychologists ([2]) and political scientists ([3]), and has been confirmed for European countries ([4]) (although there is some disagreement on the conclusion when an analysis based on time-series is used, see in particular [5] and [6]). On the other hand, life satisfaction appears to be strictly monotonically increasing with income when one studies this relation at a point in time across nations ([3]; [7]; [6]).

To reconcile the cross-sectional evidence with the Easterlin Paradox, some have suggested that the positive relation in happiness vanishes beyond some value of income ([8]; [3]; [3]; [9]). This last interpretation has been questioned by [7] and [6], who claim that there is a positive relation between GDP and life satisfaction in developed countries. From the opposite perspective, it is being questioned by [10], who provide some evidence of no long-run effect even for developing countries.

Differently from the previous literature, we perform our analysis without imposing a particular functional form to the econometric model; thus our conclusions will be independent of any hypothesis on the function linking happiness and income that we estimate. We instead partition all individual observations into quantiles of per capita GDP by the country of residence (with the 1st quantile of the distribution containing the fraction of individuals living in the poorest country), then we estimate the relation of happiness by using the quantiles. We initially consider a partition using 15 quantiles, then we repeat the analysis for partitions of 30 and 50 quantiles as a robustness check.

The second important methodological feature of our analysis is the introduction of a country-specific effect, to control for time-invariant country-specific unobservable variables, therefore eliminating a potential source of country-specific measurement errors and omitted-variable bias. The introduction of this control is of crucial importance for analysis based on survey data, because the questionnaires are generally different across countries, and there are pervasive effects due to culture and language. Many measurement errors in indices of life satisfaction are possible, for example, a well known error is the *differential item functioning*, defined as the inter-personal and inter-cultural variation in interpreting and using the response categories for the same question ([11]). [12] using vignettes to correct for Individual-Specific Scale Biases show that variations in response scales explain a large part of the cross European country differences found in raw data. If the differential item functioning generates a systematic measurement error in the life satisfaction reports, this could lead to either a positive or negative bias depending on the correlation between the measurement error and other variables in the regression. For example, if Western countries tend to over-report their life satisfaction, this could generate a positive bias in cross-country estimates of the impact of income on life satisfaction. Omitted-variable bias could be equally problematic. For example, if cultural elements determine a time invariant preference for public good supply in some country, or if income distribution – usually very persistent in time – is correlated with both life satisfaction and GDP, this would result in a bias in the relation between GDP and life satisfaction. Controlling for country specific effects eliminates all biases that could be generated by the time invariant unobservable variables mentioned in the examples. Furthermore, the panel structure of the WVS offers the possibility to include the year fixed-effect that, together with individual employment status and personal income, allows to control for the main effects of the short-run business cycles that it is well known to have an impact on life satisfaction ([9]; [10]).

[6] and [13] also estimated the effect of life satisfaction over GDP, using the WVS and controlling for country effects, but they impose a logarithmic functional form. [14] allow for the possibility of a different functional structure between rich and poor countries, but do not introduce any control for country fixed-effect (hence for countries' unobserved heterogeneity).

To further assess the importance of taking into account the unobserved heterogeneity, we perform a second analysis of the relationship between aggregate income and life satisfaction on more homogeneous territorial units. We restrict our sample to all countries within the European Union (EU) before the first enlargement (we will refer to this group of countries as the EU14) to eliminate potentially confounding factors at the country level; we perform our analysis using the European regions defined following the *Nomenclature of Territorial Units for Statistics* (NUTS2) used by the EU as a base of observation. Finally, we use the data on EU14 to investigate possible explanations of the non strictly monotonic pattern between GDP and life satisfaction.

The paper is organized as follows: Section Results first presents a broad outline of the main results, it then proceeds with a detailed presentation of the analysis, starting with country based analysis, and then following with a region based analysis. In section Discussion, we discuss possible reasons for the patterns we discovered and provide conclusions. Data are presented in section Materials.

## Results

### Overview of the Main Results

Dummy variables indicating the quantiles of the per capita GDP distribution among countries were used as the main explanatory variables. The coefficients on the quantile dummies show that life satisfaction strongly increases with GDP in low income countries, but the relation becomes much less steep beyond a GDP of 10,000 USD then it flattens for countries with a GDP above 15,000 USD. Life satisfaction shows a tendency to decline with GDP for the richest countries, suggesting the existence of a bliss point that lies in the interval between 26,000 and 30,000 2005 USD, in PPP.

In the second analysis, we focused on regional observations among the following 14 European Union countries (Austria, Belgium, Denmark, Finland, France, Germany, Greece, Italy, Luxembourg, Netherlands, Portugal, Spain, Sweden, United Kingdom), before the inclusion of the east European countries. We obtained similar results in the relation between individual life satisfaction and regional GDP. Data show a clearly positive relation between aggregate income and life satisfaction in the poorer regions, but this relation flattens and appears to turn negative for richer regions, with a bliss point between 30,000 and 33,000 2005 USD in PPP.

In our third analysis, we looked for an explanation of our previous findings. We showed with a simple example that if the relation between GDP and life satisfaction is the result of combined effects of aspirations to increase personal income, or an increasing target in terms of income comparison, then the net effect on life satisfaction is not necessarily monotonic. In [15], we provide a more micro-founded model, where income is endogenous and increases with aspirations, if the probability of fulfilling aspirations is decreasing in aspirations, this can generate a negative effect on life satisfaction that can counterbalance the positive direct effect of the income. We test this hypothesis using the EU14 data and find the usual positive effect due to the personal income and a negative effect due to the negative distance between personal income and regional GDP. Using modern personality theory, we argue that this second effect can be related to the negative effect induced by the distance from the target income. We predict that this effect should be higher for more neurotic individuals, naturally more averse to losses, and find support in the data for this explanation.

### Country Based Analysis

We started by partitioning all individual observations into 15 quantiles of about 21,000 observations each (the resulting GDP brackets of each quantile and the county-wave combinations in each bracket are presented in section S3 of File S1). A similar analysis, with the partition in 30 and 50 quantiles, is presented in section S1 of File S1.

We estimate variations of the following model:
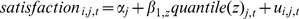
(1)where 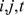 denote respectively the individual 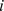, country 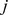 and period 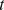. The term 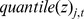 is a dummy variables equal to 1 if the country 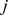 at time 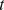 belongs to the quantile 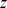 and 0 otherwise; the 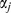
's are country dummies. For expositional simplicity, we will always consider last quintile, the one containing the richest countries, as the reference to compare all other groups and we will therefore omit it in all specifications of model (1) that will follow. In order to take into account the ordinal nature of the life satisfaction variable, we used an ordered probit estimator; to take into account the possible heteroscedasticity in the data, we clustered the errors at wave and country level to calculate the standard errors. It is perhaps useful to note that the ordered probit estimator for this model is consistent even if we are using country-specific dummies. The reason is that we are using individual data, this avoids the incidental parameters problem generated by the increase of parameters with the number of observations, 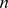, following the introduction of the individuals' fixed effects.

In table 1 we present different variations of model 1. Since we use the last quantile as the base level, a positive (negative) coefficient implies a positive (negative) differential effect on life satisfaction with respect to the last quantiles. We recall that we are controlling for country fixed-effect, hence the coefficient of the 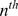 quantile can be interpreted as the effect in terms of life satisfaction of a country passing from the last to the 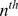 quantile. More precisely, if this coefficient is significantly negative we can say that the life satisfaction report in the country when it belongs to the 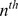 quantile is stochastically dominated by the one of the same country when it belongs to the 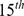 quantile. Note that the existence of positive and significant coefficients on any of the quantile dummies reveal a non monotonically-increasing pattern.

**Table 1:** GDP and life satisfaction in all WVS countries and waves. Ordered Probit Estimation.

	All	All	Exclusions	All	All
GDP				0.6602***	
				(0.1281)	
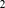 GDP				–0.1005***	
				(0.0229)	
Ln(GDP)					0.4757***
					(0.0854)
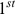 quantile	–1.5414***	–0.9139***	–0.6421**		
	(0.2019)	(0.1161)	(0.2978)		
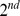 quantile	–0.9741***	–0.8906***	–0.7692***		
	(0.1512)	(0.1146)	(0.1645)		
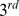 quantile	–0.9038***	–0.9118***	–0.8437***		
	(0.1464)	(0.1382)	(0.1474)		
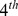 quantile	–0.5146***	–0.8000***	–0.5150***		
	(0.0991)	(0.0897)	(0.0983)		
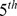 quantile	–0.4921***	–0.2881	–0.4852***		
	(0.1079)	(0.2273)	(0.1073)		
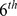 quantile	–0.4249***	–0.7808***	–0.4216***		
	(0.1023)	(0.1054)	(0.1013)		
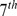 quantile	–0.2415**	–0.4291***	–0.2389**		
	(0.1035)	(0.1062)	(0.1026)		
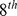 quantile	–0.1083	–0.3701***	–0.1051		
	(0.1038)	(0.0867)	(0.0991)		
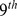 quantile	–0.0288	–0.3951***	–0.0287		
	(0.0729)	(0.1071)	(0.0724)		
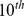 quantile	0.0169	–0.2857***	0.0170		
	(0.0502)	(0.0669)	(0.0500)		
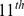 quantile	0.0317	–0.1383*	0.0317		
	(0.0370)	(0.0731)	(0.0369)		
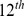 quantile	0.0844***	–0.0621	0.0842***		
	(0.0321)	(0.0590)	(0.0320)		
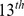 quantile	0.0389	–0.1952***	0.0388		
	(0.0306)	(0.0648)	(0.0305)		
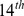 quantile	0.0726**	–0.0855	0.0724**		
	(0.0354)	(0.0714)	(0.0352)		
Country Effect	Yes	No	Yes	Yes	Yes
Wave Effect	No	No	No	No	Yes
N	307299	307299	313901	307299	307299

Dependent variable: life satisfaction. Country data refer to waves 1981–1984, 1989–93, 1994–99, 1999–04, 2005–08. Dummy of the last quantile (the 15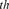) is omitted. GDP is the per capita GDP in PPP, in 10K, 2005 UDS. The countries excluded in column 3 are Luxembourg and Singapore. Standard errors clustered at country and wave levels (in brackets); *** 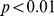, ** 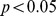, * 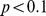.

In particular from column 1 of table 1, we note that there is a clear significantly positive differential effect (i.e. the coefficients are negative and significant) between life satisfaction of individuals living in the richest countries (with a GDP larger than 38K 2005 USD in the 15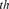 quantile) and individuals in the 7th quantile and below (i.e. individuals living in countries with less than about 10K USD). The coefficients are not statistically different from 0 within the interval 11K and 25K (between quantile 7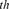 and 11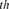), then they turn positive until the 14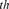 quintile. Therefore, column 1 of table 1 suggests a flattening of this relation after the 7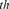 quantile and a non monotonic pattern in the last quantile.

Table 2 presents the marginal effects of the estimated model presented in the 1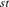 column of table 1. The 1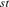 column of table 2 shows the estimated marginal effects of the different quantiles on the probability of declaring the highest level of life satisfaction, 10. The 2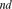 column shows the elasticities obtained by estimating an OLS model, therefore assuming a cardinal structure to the life satisfaction reports. The probability of reporting the highest level of life satisfaction is more than 10% lower in the poor countries belonging to the first three quantiles (with a GDP below 5,600 USD) than in the counties belonging to the 8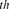 quantile (with a GDP between 13,000 and 17,000 USD). In counties above the 8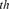 quantile (with an income above 17,000 USD), the probability of reporting the highest level of life satisfaction changes within a range of 2%. Furthermore, we note that individuals in the 12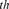 quantile (26,500 – 29,900 USD) have about 2% more chance of declaring the highest level of satisfaction than individuals in the last quantile which, again, seems to support the existence of a non monotonic pattern.

**Table 2:** Marginal effects of the GDP quantiles on life satisfaction, in the 15-quantile partition of all WVS data.

	Life satisfaction = 10	OLS
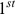 quantile	−0.1229 ***	–3.6199***
	(0.0110)	(0.4722)
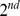 quantile	−0.10534***	–2.3267***
	(0.0109)	(0.3508)
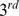 quantile	−0.1027***	–2.1651***
	(0.0114)	(0.3379)
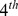 quantile	−0.0725***	–1.2369***
	(0.0138)	(0.2188)
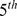 quantile	−0.0705***	–1.1868***
	(0.0194)	(0.2410)
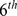 quantile	−0.0633***	–1.0284***
	(0.0163)	(0.2261)
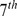 quantile	−0.0395*	–0.5903***
	(0.1377)	(0.2261)
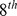 quantile	−0.0191	–0.2772
	(0.0170)	(0.2128)
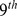 quantile	−0.0053	–0.1297
	(0.0125)	(0.1513)
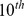 quantile	.0032	–0.0200
	(0.0083)	(0.1057)
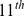 quantile	0.0060*	0.0303
	(0.0036)	(0.0717)
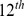 quantile	0.0165***	0.1333**
	(0.0046)	(0.0600)
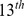 quantile	0.0074**	0.0479
	(0.0033)	(0.0580)
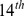 quantile	0.0141***	0.1173*
	(0.0036)	(0.0660)
Country Effect	Yes	Yes
N	307299	307299

*1^s^t column:* Elasticity of the quantile dummy variables to the probability that satisfaction  =  10, the maximum level. *2^n^d column:* column: elasticity of the quantile dummy variables estimated using a linear model (OLS with country specific effect). The base level is the last quantile (the 15^th^), grouping the countries with per capita GDP larger than 36.81K. The coefficients are derived from the estimation of the baseline specification of model (1). GDP is reported in 10K, 2005 USD, PPP adjusted. Standard errors are clustered at country and wave levels (in brackets); *** p<0.01, ** p<0.05 , * p<0.1.

*1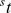 column:* Elasticity of the quantile dummy variables to the probability that satisfaction  =  10, the maximum level. *2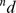 column:* elasticity of the quantile dummy variables estimated using a linear model (OLS with country specific effect). The base level is the last quantile (the 15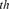), grouping the countries with per capita GDP larger than 36.81K. The coefficients are derived from the estimation of the baseline specification of model (1). GDP is reported in 10K, 2005 USD, PPP adjusted. Standard errors are clustered at country and wave levels (in brackets); ***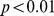, ** 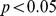, * 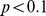.

These results are consistent with those in column 4 of table 1, where we imposed a quadratic structure to the estimated model, whose interpolating line reach its peak at about 31K (statistically different from the upper bound of 64K). Comparing the 1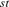 with the 2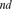 column of table 1, we note that the relationship between life satisfaction and country GDP seems strictly monotonic when we do not include the country specific effect; this is consistent with the current literature (e.g. [7]; [6]). In column 5, we present a logarithmic specification specification similar to the one in table 3 of [13], with a logarithmic model and the wave fixed effect. The coefficient we find is close to 0.5, the one they find.

**Table 3:** Regional GDP and life satisfaction in EU14 regions.

	EU14	EU14	EU14	EU14	EU14
Reg.GDP					0.3041***
					(0.0335)
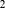 Rge.GDP					–0.0320***
					(0.0037)
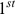 quantile	–0.1366***	–0.0896***	–0.0929***	–0.0502***	
	(0.0022)	(0.0204)	(0.0217)	(0.0188)	
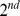 quantile	–0.1153***	–0.0627***	–0.1682***	–0.1424***	
	(0.0018)	(0.0118)	(0.0237)	(0.0256)	
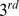 quantile	–0.0702***	–0.0093	–0.0785***	–0.0583***	
	(0.0008)	(0.0238)	(0.0078)	(0.0102)	
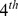 quantile	0.0594***	0.0361***	0.0733***	0.1045***	
	(0.0013)	(0.0097)	(0.0095)	(0.0140)	
Income Step 2				0.0669*	
				(0.0390)	
Income Step 3				0.1806***	
				(0.0391)	
Income Step 4				0.2944***	
				(0.0295)	
Income Step 5				0.3494***	
				(0.0354)	
Income Step 6				0.4181***	
				(0.0377)	
Income Step 7				0.5705***	
				(0.0528)	
Income Step 8				0.5696***	
				(0.0520)	
Income Step 9				0.5930***	
				(0.0473)	
Income Step 10				0.6697***	
				(0.0470)	
Age			–0.0080**	–0.0185***	–0.0067*
			(0.0038)	(0.0042)	(0.0035)
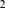 Age			0.0001**	0.0002***	0.0001**
			(0.0000)	(0.0000)	(0.0000)
Male			–0.0173	–0.0341	–0.0122
			(0.0229)	(0.0234)	(0.0164)
Education	No	No	Yes	Yes	No
Employment Status	No	No	Yes	No	No
Year Effect	No	No	Yes	No	No
Town Size	No	No	Yes	Yes	No
Country Effect	No	Yes	No	No	No
N	32091	32091	23623	18192	31994

Ordered Probit Estimation. Data refer to waves 1994–99, 1999–04, 2005–08. Dummy of the last quantile (the 5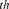) is omitted. Reg.GDP is the per capita regional GDP in PPP, in 10K, 2005 USD. Standard errors are clustered at country and wave levels; *** 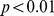, ** 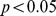, * 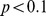.

The countries belonging to the last quintile are: Australia, Luxembourg, Netherlands, Norway, Singapore, Sweden, Switzerland, UK, US; each represented in one or more waves (in section S3 of File S1, we can observe the precise country wave combinations belonging to each quantile). The non monotonic relation is robust to the exclusion of Singapore and Luxembourg, as we can see from the 3rd column of table 1.

In section S1 of File S1 we show the estimation results of a more complete specification of model 1. In particular, we show that our results are robust to the introduction of yearly fixed-effect, individual demographic, education, employment status, and personal income. It is therefore arguable that the relation between aggregate incomes and life satisfaction is due to external effects.

### Region Based Analysis

We showed that when one controls for country heterogeneity by introducing a country specific effect, life satisfaction does not seem to be monotonically increasing with GDP. In order to validate the former result we now analyze the relation between GDP and life satisfaction among more homogeneous territorial units. We restrict our selection to all countries belonging to the EU14, in order to have more variation and observations we consider the data at regional level (this information is present in the WVS dataset for European Countries. We could not perform a similar exercise using US observations in the WVS since there is no indication of the state the individual belongs to, and data can only be decomposed in 4 macro-regions). Given the higher level of homogeneity within the group of countries we are considering, we expect that a relation similar to the one we have seen holds for this selection, without controlling for country (or region) effects.

In a similar way as before we group observations into 5 quantiles based on GDP for each region-wave, with about 6,500 observations per quantile in the 5 quantiles (in section S4 of File S1, we show the list of region-wave per quantile). In table 3 we present the results, with data partitioned in 5 quantiles, as before the last quantile is the base one, and for this reason it has been omitted. Given the small amount of observations in several regions we calculated the standard errors by clustering the errors at quantile levels.

Column 1 of table 3 shows that life satisfaction invariably increases in the first 4 quintiles and decreases in the last. In this column – consistently with the logic of this second test – we are not controlling for country, or regional heterogeneity. In column 2 of table 3 we show that the result is robust to the introduction of country fixed-effect. In column 3 we introduce town size dummies to control for congestion (given that several regions are in fact constituted by a large city), employment status, education and yearly fixed effect. Note that the year fixed effect is a particularly effective control for economic cycles given the high degree of economic integration among the European regions in the sample. Finally, in column 4 of table 3 we observe that the non monotonic relation between regional GDP and life satisfaction is robust to the introduction of individual income.

**Figure 1:**
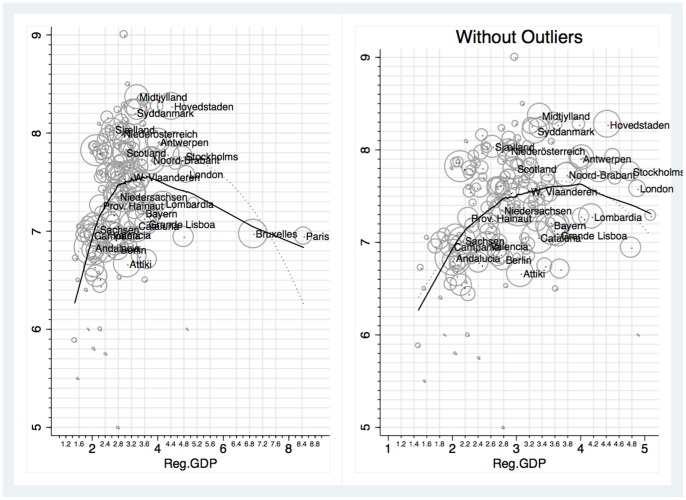
Average life satisfaction and aggregate Incomes in EU14 Regions. A circle in the scatter plot represents the regional average life satisfaction and average regional GDP. Both variables are averages pooling together the waves 1994–99, 1999–2004 and 2005–08. The weights are the sample sizes for each region. The continuous line represents the Lowess function, the dotted line is the quadratic interpolation, where data are weighted by the sample size. The equation in the left panel is: 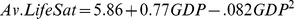 with 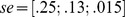. The equation in the right panel is 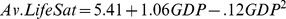 with 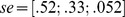 Per capita regional GDP measures are in 10K 2005 USD and are PPP adjusted.

This non monotonic pattern can be observed from Figure 1. We aggregated all waves for which the information on regional residence and regional GDP were available, i.e. waves 1994–99, 1999–2004, 2005–08. The solid line in both panels of Figure 1 represents the Lowess function, which displays for each value of the independent variable (Regional GDP) a smoothed value of the dependent variable (average life satisfaction). The dotted lines are the quadratic interpolations. Both the linear and quadratic coefficients of the quadratic interpolations are highly significant and consistent with a peak internal to the regional GDP intervals. Note that in the panel without outliers, the estimated Lowess function follows the quadratic interpolation closely.

Figure 1 may suggest that the declining segment of the curve is due to only two observations, Brussels and Paris. We repeated all regressions in table 3 excluding these two observations; results are presented in table 4. From column 1 we note that the coefficient of the 4th quantile dummy is still significantly positive, although smaller in magnitude. Hence the non monotonic relation between regional GDP and life satisfaction is robust to this exclusion. In column 2 to 4 we added more controls, finding similar results (although in one case the coefficient of the 4th quantiles looses its statistical significance). In section S1 of of File S1 we repeat the above analysis with a 10 quantile partition.

**Table 4:** Regional GDP and life satisfaction in EU14 regions without Brussels and Paris.

	no outliers	no outliers	no outliers	no outliers
*GDP in*				
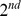 quintile	0.3004***	0.1825***	0.2406***	0.1886***
	(0.0000)	(0.0000)	(0.0000)	(0.0000)
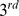 quintile	0.6944***	0.3913***	0.5641***	0.5437***
	(0.0000)	(0.0000)	(0.0000)	(0.0000)
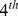 quintile	0.7246***	0.3708***	0.4646***	0.6200***
	(0.0000)	(0.0000)	(0.0000)	(0.0000)
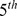 quintile	0.9244***	0.5809***	0.6780***	0.7798***
	(0.0000)	(0.0000)	(0.0000)	(0.0000)
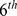 quintile	1.1846***	0.6243***	0.9332***	1.0370***
	(0.0000)	(0.0000)	(0.0000)	(0.0000)
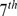 quintile	1.2412***	0.7613***	1.1113***	1.1003***
	(0.0000)	(0.0000)	(0.0000)	(0.0000)
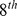 quintile	1.2720***	0.9075***	1.1048***	1.0845***
	(0.0000)	(0.0000)	(0.0000)	(0.0000)
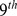 quintile	1.2696***	0.8189***	1.0740***	1.0809***
	(0.0000)	(0.0000)	(0.0000)	(0.0000)
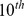 quintile	1.2331***	0.9851***	1.0512***	1.0687***
	(0.0000)	(0.0000)	(0.0000)	(0.0000)
Income Steps				Yes***
Age			Yes	Yes
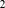 Age			Yes	Yes
Male			Yes	Yes
Education	No	No	Yes	No
Employment status	No	No	Yes	No
Country Effect	Yes	No	Yes	Yes
Year Effect	No	No	Yes	Yes
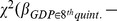	16.73***			
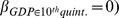	(0.0000)			
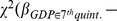			6.47**	1.82
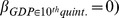			(0.011)	(0.178)
N	305124	305124	224337	258847

Ordered Probit Estimation. Data refer to waves 1994–99, 1999–04, 2005–08. Dummy of the last quantile (the 5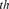) is omitted. Reg.GDP is the per capita regional GDP in PPP, in 10K, 2005 USD. Standard errors are clustered at quantile level (in brackets); ***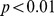, ** 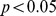, * 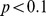.

### A Simple Model of Aspiration

How can this non monotonic pattern be explained? A simple example may clarify this issue. Assume that individual life satisfaction is combining an increasing and concave utility function 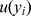 of the personal income 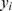 with a negative function depending on the difference between personal income and target level 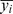. So Life Satisfaction LS is:
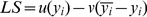
(2)where 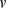 is an increasing function. The value 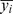 can reflect the income of a reference group (i.e. the “Joneses”), or to an aspiration level for individual 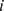. For expositional simplicity, assume that the majority of individuals have the same personal income, and let it be fraction, 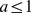 of the per capita GDP, 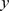. They have the same level of aspiration 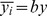, with 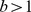, so that the target income for most individuals is increasing with GDP. Therefore, even if the personal income increases with the GDP, the level of life satisfaction can be non monotonic in GDP, 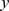 (using the simple functional form: 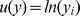 and 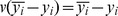. The life satisfaction will be hump shaped with a peak in 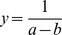).

To check the existence of this effect in our data, we estimate a model based on the EU14 regional data, where life satisfaction depends on the logarithm of personal income, the logarithm of Regional GDP, the difference between personal income and regional GDP, as well as other individual and country specific control variables. Results are reported in table 5 (considering the Logarithm of the differences does not qualitatively effect the result of the estimation). In the WVS, data on household income is expressed in 10 or 11 country-specific brackets we derived the personal income variable by taking the middle value of each bracket, and then transforming the data into adjusted 2005 USD PPP. The summary statistics of the derived personal income variable, in 10,000 USD, are presented in section S2 in File S1.

**Table 5:** Individual income, per capita GDP and life satisfaction in EU14.

	EU14	EU14	EU14	EU14
ln(Income)	0.1908***	0.2379***	0.1678***	0.1293***
	(0.0421)	(0.0282)	(0.0379)	(0.0482)
ln(Reg.GDP)	0.1390*	–0.0067	0.1719**	0.5141***
	(0.0831)	(0.0660)	(0.0820)	(0.1284)
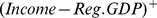	–0.0093			0.0082
	(0.0102)			(0.0142)
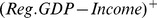	–0.0508**		–0.0600**	–0.1355***
	(0.0246)		(0.0239)	(0.0316)
Dummy(Income 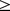 Reg.GDP)	0.0128	0.0061	0.0087	–0.0007
	(0.0300)	(0.0310)	(0.0299)	(0.0341)
Age	–0.0195***	–0.0188***	–0.0194***	–0.0170***
	(0.0035)	(0.0035)	(0.0035)	(0.0055)
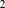 Age	0.0002***	0.0002***	0.0002***	0.0002***
	(0.0000)	(0.0000)	(0.0000)	(0.0001)
Male	–0.0247	–0.0232	–0.0246	–0.0469**
	(0.0157)	(0.0156)	(0.0157)	(0.0184)
Unemployed	–0.5258***	–0.5324***	–0.5265***	
	(0.0588)	(0.0583)	(0.0587)	
Country Effect	Yes	Yes	Yes	No
Town Size	Yes	Yes	Yes	No
N	15585	15585	15585	17392

Ordered Probit Estimation. Dependent variable is individual life satisfaction; data refer to waves 1994–99, 1999–04, 2005–08, townsize includes dummy variables controlling for 8 different town sizes. Per capita regional GDP and personal income is in 10K 2005 USD and is PPP adjusted. 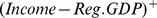 is set to 0 if 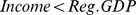, and 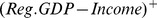 is set to 0 if 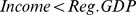. Standard errors are clustered at regional level (in brackets); ***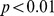, ** 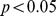, * 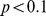.

Now, we introduce the difference between personal income and regional GDP separately as a positive difference, 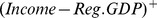 (equal to 0 if 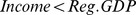) and negative difference 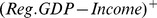 (equal to 0 if 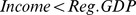). The term 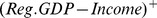 is a proxy for the difference 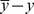 as defined above. Given that the median income is generally smaller than the average income, for the majority of the population 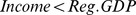. This is consistent with the observations in our sample, where we have 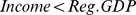 for about 61% of the observations. We therefore expect this term to be negative with respect to life satisfaction; table 5 confirms this prediction. We note that life satisfaction is negatively correlated with the difference between regional and personal income, when this difference is negative. At the same time the positive difference does not significantly affect life satisfaction.

The asymmetry between positive and negative differences suggests an interpretation attributing a larger impact to losses than gains. In this respect, the data in the WVS allows to perform a further test using modern studies on personality theory (see [16] for a recent survey). In particular neuroticism has been recently associated with higher sensitivity to negative emotions like anger, hostility or depression ([17]) and with structural features of the brain system associated with sensitivity to threat and punishment ([18]). Neuroticism also signals low levels of serotonin, which in turn is associated with aggression, poor impulse control, depression, and anxiety ([19]; [20]). Neuroticism is identified with sensitivity to negative outcomes. Therefore, we suggest that the elasticity between individual life satisfaction and 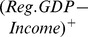 could be modulated by Neuroticism.

Measures of personality traits are not available in the WVS, but using the standard procedure of performing factor analysis on all the 20 personality questions, available in wave 1989–93 of the WVS data-set, we determine the personality traits Neuroticism and Extraversion. Details on the way Neuroticism and Extraversion have been generated and the list of the personality questions are presented in section S5 of File S1, together with summary statistics of the Neuroticism and Extraversion variables obtained in this way. Table 6 confirms our prediction that elasticity between individual life satisfaction and 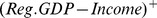 is modulated by Neuroticism. 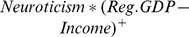 is negative and significant suggesting a stronger negative effect of the difference 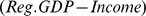, for more neurotic individuals. Note that our derived personality traits affect life satisfaction in a way consistent with findings of the literature using measures of personality derived by surveys: It is a well known finding that extraversion is positively correlated with life satisfaction and the opposite is true for Neuroticism. Furthermore in [15] we find consistent results using the British Household Panel and the German Socioeconomic Panel Surveys, where personality traits are determined using a standard questionnaire.

**Table 6:** Individual income, per capita GDP and life satisfaction in EU14.

	EU14	EU14	EU14	EU14
ln(Income)	0.0773	0.0685	0.0643	0.0549
	(0.0576)	(0.0556)	(0.0536)	(0.0507)
ln(Reg.GDP)	0.3426*	–0.0045	0.3198	–0.0349
	(0.1997)	(0.1881)	(0.2040)	(0.1933)
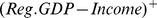	–0.1259***	–0.0424	–0.1455***	–0.0652
	(0.0470)	(0.0453)	(0.0419)	(0.0401)
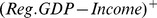 Neurot*	–0.0320**	–0.0321**	–0.0355**	–0.0360**
	(0.0147)	(0.0143)	(0.0150)	(0.0149)
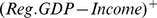 Extr*		0.0020		–0.0018
		(0.0145)		(0.0146)
Dummy (Income 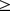 Reg.GDP)	–0.1736*	–0.1450*	–0.2186**	–0.1978**
	(0.0898)	(0.0815)	(0.0876)	(0.0792)
Neuroticism	–0.4094***	–0.4302***	–0.4016***	–0.4211***
	(0.0463)	(0.0462)	(0.0471)	(0.0477)
Extraversion		0.2834***		0.2886***
		(0.0411)		(0.0415)
Age	–0.0118***	–0.0089***	–0.0115***	–0.0087**
	(0.0033)	(0.0034)	(0.0034)	(0.0034)
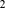 Age	0.0001***	0.0001***	0.0001***	0.0001***
	(0.0000)	(0.0000)	(0.0000)	(0.0000)
Male	–0.0657***	–0.0900***	–0.0664***	–0.0905***
	(0.0206)	(0.0209)	(0.0208)	(0.0211)
Unemployed	–0.4358***	–0.3989***	–0.4415***	–0.4077***
	(0.0440)	(0.0430)	(0.0433)	(0.0421)
Town Size	Yes	Yes	No	No
	10492	10492	10521	10521

Ordered Probit Estimation. Dependent variable is individual life satisfaction; data refer to wave 1996–06, townsize includes dummy variables controlling for 8 different town sizes. Per capita regional GDP and personal income is in 10K 2005 USD and is PPP adjusted. 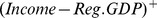 is set to 0 if 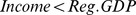, and 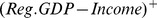 is set to 0 if 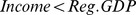. Standard errors are clustered at regional level (in brackets); ***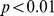, ** 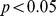, * 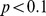.

## Discussion

We have reexamined the relationship between life satisfaction and GDP without imposing a particular functional form and found robust evidence of a clearly increasing relationship among poor countries and a non monotonic relation for richer countries. This finding lends support to the idea that the conflict between cross-sectional evidence – showing a positive relationship between GDP and life satisfaction – and the times-series evidence – generally finding no relationship – can be reconciled if the positive effect of GDP disappears after some bliss point ([8]; [3]; [21]; [9]). Furthermore, our analysis shows evidence of a non monotonic relationship between GDP and life satisfaction toward the end of the spectrum among the richest countries, with Life satisfaction slightly decreasing after a bliss point.

Our findings on the relationship between GDP and life satisfaction are not in contrast with the previous cross sectional analysis. The differences with this literature are easily explained by the method we use; we replicated the results in the cross-country based literature when similar methods are used. We found a strictly monotonic relation between GDP and life satisfaction if we do not introduce country-specific dummies (in column 2 of table 1). We also replicated the results of [13], who estimated the effect of life satisfaction over GDP by using the WVS and controlling for country effects with a logarithmic model (in column 5 table 1). Similarly, our findings are not in contrast with the previous times-series based analysis, mostly focused on developed countries, but it allows us to pool data to an extent which is larger than what is allowed by separate times-series analysis at country level.

Such non-monotonicity of the relationship suggests the need for a new way of thinking about this relationship, which we think has independent interest, and provides a bridge between existing economic theory and richer, although more informal, theories of human behavior like Personality Theory. Therefore, we investigated the reasons for the non-monotonic relationship. It is well known that life satisfaction is increasing in personal income at a decreasing rate (e.g. [22]). [23] find that the marginal life satisfaction with respect to income declines at a rate faster than the one implied by a logarithm utility function. This finding is substantially supported by [24] who argue, using USA data, that the effect of income on the emotional dimension of well-being is strictly increasing until an annual income of 75,000 USD, but has no further positive influence for higher values. However, a considerable literature following the Easterlin paradox suggest that this link is complicated by the existence of other effects acting with an opposite sign. The first is that the aspirations adapt to the new situations, an idea originally proposed by [25] and recently reassessed by [26]. [27], [28], [29], [30] provide some empirical evidence on how aspirations increase in income. The second is the effect of the relative income on individual life satisfaction – the so-called “Keeping up with the Joneses” hypothesis – an idea that can be dated back to [31]. [32], [22], [33], [34], [35] among others present empirical validations of this hypothesis ([17] provide an extensive survey of the theoretical and empirical literature explaining the Easterlin Paradox).

In the view we propose, higher GDP leads to higher aspirations (driven by the existence of more opportunities or by comparison with the *Joneses*), which drives effort and individual commitment, which in turn do, on average, produce higher income. This higher income would typically produce higher life satisfaction. If we did stop here we would predict higher income to be associated with higher life satisfaction, perhaps at a decreasing rate. However, higher income now sets up a race between aspiration and realization; when realization is lower than aspiration, the psychological cost paid is disappointment, which increases with this gap (see [2, Unpublished Data Section], for a formal characterization and a structural estimation of this model using the British Household Panel and the German Socioeconomic Panel Surveys). If we again only look at the relationship between income and life satisfaction, we might observe a non-monotonic relationship for higher incomes. Indeed, with a simple example we have shown that if the relationship between life satisfaction and GDP is the result of combined effects of aspiration, realized personal income, and disappointment, the net effect may be non-monotonic.

Our tests give support to the idea of a positive effect due to personal income and a negative effect due to the negative distance between personal income and regional GDP. This view implies that since the negative effect on happiness is induced by disappointment, this effect should be stronger in individuals who are more sensitive to losses and pay a higher psychological cost for the disappointment, which is another prediction that we test. The measure that we used of this sensitivity is the neuroticism score. In the data, we found that the way in which the relationship between personal income and life satisfaction is affected by Neuroticism is consistent with this interpretation. Individual welfare is affected by the gap between realized and desired income. When the gap is negative, for lower level of income, extra income decreases in absolute terms this negative gap; therefore individuals with higher Neuroticism score, that are more sensitive to reduced negative outcomes, become more satisfied.

Our analysis implies that GDP long term growth is certainly desirable among poorer countries, but is it a desirable feature among developed countries as well? Recent evidence provided by [36] shows the negative effect of high aspiration can also be rationally predicted by individuals that, nevertheless may still choose options that do not always maximize happiness, but which are compatible with high income aspirations. This implies that individuals may still prefer to live in richer countries, even if this would result in a decreased level of life satisfaction. In other words, the fact that individuals aspire to a higher income may not be considered, from an individual perspective, a negative feature of an economy even if this might result in a lower level of reported life satisfaction among the richest countries. Finally, it is perhaps worth noting that our correlations between indices of well being and indices of aggregated wealth does not necessary imply a causality relation running from GDP to life satisfaction. This relationship is indeed very complex, both the presence of omitted variables and the existence of reverse causality, as recent contributions ([37] and [38]) have emphasized, which cannot be excluded.

## Materials

We used World Values Survey (WVS) dataset (and the integrated European Value Survey) for the country based analysis, and the European Values Survey in the European region based analysis. The data are generally available for five waves: 1981–1984, 1989–93, 1994–99, 1999–04, 2005–08. We consider all available country-wave observations, excluding a few country-waves explicitly considered not representative in the WVS (the country waves excluded are Argentina, 1981–1984, 1989–93, 1999–04; Bangladesh, 1999–04; Chile, 1989–93, 1994–99; China, 1989–93, Dominican Republic, 1994–99; Egypt, Arab Rep. 1999–04; India 1989–93; Mexico, 1989–93; Nigeria, 1989–93; Pakistan 1999–04; South Africa 1989–93). The list of the country-waves and the number of observations per country-wave are presented in section S3. The dataset is repeated cross-section (i.e. individuals in the sample are different in each wave).

In the WVS, the variable used to measure personal satisfaction is the answer to the question: *“All things considered, how satisfied are you with your life as a whole these days?”* coded on a scale from 1 (dissatisfied) to 10 (satisfied). From the WVS we also derive the personal income measure, generally coded in 10 steps (and for a few country in 11 steps). The income ladder is provided as a common variable in the WVS, and it is derived by income ladders specific to each countries. Education, measured by age of leaving education, is ordinally coded from 1 to 10, ranging from less than 12 years old of age until to more than 21 years old. The categories for employment status are: full time, part time, self-employed, retired, housewife, student, unemployed, other. Town size is coded from 1 to 8, ranging from less than 2000 until 500,000 and more.

The country-level per capita GDP is from the World Bank World Development Indicators dataset, and they are in constant 2005 US international dollars, PPP adjusted. In Table 5 and 6 of section S2 of File S1, we present a description of the main variables. Data are partitioned in 15 quantiles according to the per capita GDP level (in the Section S1 of File S1 we repeat the analysis with 30 and 50 quantile partitions). The resulting GDP brackets of each quantile and the county-wave combinations in each bracket are presented in section S3 of File S1.

The European regions are defined following the *Nomenclature of Territorial Units for Statistics* (NUTS2) used by the EU; we have data for 171 regions. The regional per capita GDP data are from the Eurostat dataset; the values in Euros are PPP adjusted. We then transform the regional GDP data into constant 2005 USD, by using the Consumer Price Index (CPI) from the World Bank-World Development Indicators dataset (in a few cases the WVS regional classification did not match exactly the EUROSTAT classification, so we needed to aggregate some of the WVS regions, details are available upon request). A list of the region-wave combinations in each quantile for the 5 quantile partition is available in section S4 of File S1.

In the third analysis, aimed to investigate the reason of the non monotonic pattern unveiled in the country and region based analysis, we derived the personality traits from some personality questions present in the 1989–93 wave (this exercise is presented in the section S5 of File S1). For this reason, this analysis only used data from this wave. In section S2 of File S1 we provide a description of the main variables used in the three analysis performed in the paper.

## Supporting Information

File S1 **Supporting Information.**
(PDF)
