# Supplementary material for: A Reassessment of the Relationship between GDP and Life Satisfaction
Source: PLoS One. 2013 Nov 27;8(11):e79358. doi: 10.1371/journal.pone.0079358 (PMC3842267; doi:10.1371/journal.pone.0079358)
Supplement: File S1 — Supporting Information. (PDF) [file pone.0079358.s001.pdf]

Supporting Information

S1: Additional Analysis

This section provide a robustness check of the analysis performed in Section . In particular, we add more control variables to model 1 and we partition the countries in a larger number of smaller groups.

We start by estimating different variations of the following model:

$$satisfaction_{i,j,t} = \alpha_j + \beta_{1,z} quantile(z)_{j,t} + \eta_t + \Gamma K_{i,j,t} + u_{i,j,t} \tag{2}$$

where i, j, t denotes the individual i, country j and period t respectively. The term $quantile(z)_{j,t}$ is a dummy variables equal to 1 if the country j at time t belongs to the quantile z and 0 otherwise; α_j are country dummies, η_t are period dummies, $K_{i,j,t}$ is a vector of individual characteristics and $u_{i,j,t}$ is an error term.

Country Based Analysis

In table 1 we show the estimation results of model 2 with a 15 quantile partition like in the main text. We note that the non-monotonic pattern holds when we introduce controls for yearly effect, individual demographic (column 1), and education and employment status (column 2). Note that the introduction of personal income (in column 3) does not seem to qualitatively change the non-monotonic relationship between GDP and life satisfaction. It is therefore arguable that the relation between aggregate incomes and life satisfaction is due to external effects. From the 4th column of table 1, we note that data are consistent with a quadratic model, where the interpolating line has a maximum at around 30K, similar to the one in column 4 of table 1.

In order to have a better description of the pattern governing the relation between GDP and life satisfaction, especially in the richest countries, and also to check the robustness of the results obtained in the main text, we estimate model (1) using smaller partitions and hence grouping the data in a larger number of quantiles. In table 2, we show the coefficients of the 30 quantiles in model (1) and their confidence intervals, where errors are clustered at country and wave levels. We can therefore observe their statistical difference from 0, the base coefficient indicating the 30th quintile. Note that all coefficients between 21st and 28th quantiles are above the one in the 30th. Also this pattern seems non-monotonic,

with the coefficient increasing until the 23rd quantile – corresponding to a GDP interval of 25K-26K– and then decreasing. Figure 1 plots the estimated quantile coefficients presented in the first column of table 2.

Furthermore, we repeat the above exercise by partitioning the country-wave observations into 50 quantiles. We present the resulting quantiles' coefficients in Figure 2 and we interpolate a cubic line. The relationship is clearly monotonic until the 25th quantile, then it flattens for richer countries. The quadratic and cubic coefficients of the interpolating line are both significant at 1% level, and we can observe a maximum around the 40th quantile, corresponding to a GDP interval 28.3K- 2.8.5K. In Figure 3, we display only the coefficients of the 25 richest quantiles, corresponding to the top 50% GDP, and its quadratic interpolation with the 95% confidence interval. We observe that the quadratic interpolation features a peak at the 40th quantile; from a visual inspection of the figure we note that a monotonic pattern within the 95% confidence interval can be rejected.

Region Based Analysis

In table 4, we partitioned the regional data into 10 quantiles to check the robustness of the results for a different partition of the analysis presented in table 3 of the main text. From column 1, we note that there is an increasing positive effect until the 7th quantile, then the coefficients of the quantile dummies decrease. However, this is true until the 9th quantile since the coefficient of the 9th quantile is negative, reversing the decreasing pattern. Column 2 and 3 show that this reversion at the last quantile disappear when we control for either town size or country effect (in the top panel of Figure 4, we display the value of the coefficient of of the 10 quantile dummy relative to the estimation of column 2). We note a pattern that seem monotonically increasing (apart for the exception of the 2nd quantile) until the 7th quantile, then it is decreasing. This suggest a hump shaped pattern with a maximum in the 7th quantile, corresponding to a regional GDP within the interval 30K-33K. Columns 4 and 5 finally show that the non monotonic pattern is robust to the introduction of a number of individual controls as in the previous table.

In order to check how much of the above results are dependent from the outliers we observed in Figure 1 of the main text , we repeated the analysis above by excluding the 10th quantile (containing both Paris and Brussels) and using the 9th as base level. Results are displayed in table 3 and in the bottom panel of Figure 4, where we note a similar pattern in the analysis with all 10 quantiles; the pattern is generally increasing in the first 7 quantiles, then decreasing.

Table 1. GDP and life satisfaction in all WVS countries and waves. Ordered Probit Estimation

	All Country 1981-2006 b/se	All Country 1981-2006 b/se	with Exclusions 1981-2006 b/se	All Country 1981-2006 b/se
GDP				0.5664***
				(0.1317)
GDP^2				–0.0826***
				(0.0212)
7th quantile	–0.2512*	–0.4715***	–0.1867	
	(0.1434)	(0.1297)	(0.1651)	
8th quantile	–0.1624	–0.4437***	–0.0062	
	(0.1555)	(0.1350)	(0.1731)	
9th quantile	–0.0019	–0.1581	0.1027	
	(0.1216)	(0.1001)	(0.1384)	
10th quantile	0.0765	0.0877	0.1204	
	(0.0896)	(0.0782)	(0.0932)	
11th quantile	0.0916	0.0720	0.1609*	
	(0.0803)	(0.0792)	(0.0888)	
12th quantile	0.1032*	0.1010*	0.0921	
	(0.0586)	(0.0560)	(0.0610)	
13th quantile	0.0659	0.0519	0.0681	
	(0.0493)	(0.0483)	(0.0500)	
14th quantile	0.0880**	0.0915**	0.1083**	
	(0.0432)	(0.0409)	(0.0478)	
1st to 7th quantile	Yes	Yes	Yes	No
2nd to 11th Income Steps	No	No	Yes	No
Education	No	Yes	No	No
Employment status	No	Yes	No	No
Country Effect	Yes	Yes	Yes	Yes
Year Effect	Yes	Yes	Yes	Yes
age, age^2, Male	Yes	Yes	Yes	Yes
N	298479	226419	260393	298479

Dependent variable: life satisfaction. Country data refer to waves 1981-1984, 1989-93, 1994-99, 1999-04, 2005-08. Dummy of the last quantile (the 15th) is omitted. Emplostat represents dummies variables for: Unemployed, Full time, Part time, Self Employed, Retired, House-Keeper. Education is a series of 10 dummies controlling for different years of schooling. GDP is the per capita GDP in PPP, in 10K, 2005 USD. Standard errors are clustered at country and wave level (in brackets).

Table 2. GDP and life satisfaction in all WVS countries and waves. Ordered Probit Estimation in the 30-quantile partition.

	All Country 1981-2006 b/se	All Country 1981-2006 b/se	All Country 1981-2006 b/se	All Country 1981-2006 b/se
15th quantile	–0.3024***	–0.2229	–0.6488***	0.8329***
	(0.1048)	(0.1726)	(0.1722)	(0.1437)
16th quantile	–0.0075	–0.0438	–0.4098**	1.0151***
	(0.1198)	(0.1842)	(0.1760)	(0.1814)
17th quantile	–0.0368	–0.0054	–0.2822*	1.0287***
	(0.1286)	(0.1648)	(0.1631)	(0.1695)
18th quantile	–0.0025	0.0551	–0.1172	1.0786***
	(0.0773)	(0.1416)	(0.1330)	(0.1580)
19th quantile	0.0049	0.1003	0.1081	1.1323***
	(0.0778)	(0.1185)	(0.1194)	(0.1841)
20th quantile	0.1014	0.1305	0.0860	1.0353***
	(0.0749)	(0.1095)	(0.1005)	(0.1857)
21st quantile	0.1369***	0.2016*	0.1060	1.1982***
	(0.0529)	(0.1092)	(0.1114)	(0.1905)
22th quantile	0.0671	0.1076	0.0723	1.0791***
	(0.0563)	(0.0960)	(0.0974)	(0.1964)
23rd quantile	0.1804***	0.1453*	0.1038	1.0372***
	(0.0545)	(0.0854)	(0.0802)	(0.2038)
24th quantile	0.1080**	0.1261*	0.0870	1.0313***
	(0.0486)	(0.0737)	(0.0708)	(0.2071)
25th quantile	0.1150**	0.0765	0.0388	1.0004***
	(0.0454)	(0.0680)	(0.0670)	(0.2097)
26th quantile	0.1044*	0.1063	0.0722	1.0229***
	(0.0539)	(0.0681)	(0.0724)	(0.2100)
27th quantile	0.1536**	0.1346**	0.0844	1.0838***
	(0.0613)	(0.0676)	(0.0667)	(0.2122)
28th quantile	0.1083**	0.0936	0.0940	1.0459***
	(0.0486)	(0.0609)	(0.0574)	(0.2188)
29th quantile	0.0913*	0.0457	0.0266	0.9487***
	(0.0524)	(0.0609)	(0.0574)	(0.2222)
1st to 14th quantile	Yes	Yes	No	Yes
2nd to 11th Income Steps	No	No	No	Yes
Education	No	No	Yes	No
Employment status	No	No	Yes	No
Country Effect	Yes	Yes	Yes	Yes
Year Effect	No	Yes	Yes	Yes
age, age^2, Male	Yes	Yes	Yes	Yes
N	298479	298479	226419	260393

Dependent variable: life satisfaction. Country data refer to waves 1981-1984, 1989-93, 1994-99, 1999-04, 2005-08. Dummy of the last quantile (the 15th) is omitted. Emplostat represents dummies variables for: Unemployed, Full time, Part time, Self Employed, Retired, House-Keeper. Education is a series of 10 dummies controlling for different years of schooling. GDP is the per capita GDP in PPP, in 10K, 2005 USD. Standard errors are clustered at country and wave level (in brackets).

Table 3. Regional GDP and life satisfaction in EU14 regions without regions in the 10th quantile

	EU14<br>1996-2006<br>b/se	EU14<br>1996-2006<br>b/se	EU14<br>1996-2006<br>b/se	EU14<br>1996-2006<br>b/se	EU14<br>1996-2006<br>b/se
1st quantile	–0.2607***	–0.3273***	–0.1226***	–0.1429***	–0.1400***
	(0.0042)	(0.0118)	(0.0130)	(0.0179)	(0.0293)
2nd quantile	0.0274***	–0.0541***	–0.0557***	–0.0752***	–0.0593***
	(0.0021)	(0.0078)	(0.0149)	(0.0159)	(0.0093)
3rd quantile	–0.1396***	–0.2738***	–0.1170***	–0.2113***	–0.1487***
	(0.0027)	(0.0131)	(0.0127)	(0.0209)	(0.0254)
4th quantile	–0.0636***	–0.1343***	–0.0444***	–0.1544***	–0.0684***
	(0.0018)	(0.0094)	(0.0159)	(0.0156)	(0.0111)
5th quantile	–0.0747***	–0.1025***	–0.0644***	–0.0919***	–0.0335**
	(0.0017)	(0.0038)	(0.0176)	(0.0135)	(0.0144)
6th quantile	–0.0386***	–0.0539***	0.0190	–0.0812***	–0.0365**
	(0.0015)	(0.0056)	(0.0241)	(0.0142)	(0.0142)
7th quantile	0.0997***	0.0619***	0.0150	0.0773***	0.1028***
	(0.0020)	(0.0040)	(0.0104)	(0.0092)	(0.0110)
8th quantile	0.0471***	0.0321***	0.0367***	0.0508***	0.1386***
	(0.0017)	(0.0034)	(0.0111)	(0.0102)	(0.0085)
Age				–0.0063**	–0.0160***
				(0.0026)	(0.0028)
Age^2				0.0001***	0.0002***
				(0.0000)	(0.0000)
Male				–0.0237	–0.0220
				(0.0262)	(0.0289)
Education	No	No	No	Yes	No
Employment Status	No	No	No	Yes	No
Year Effect	No	No	No	Yes	Yes
Town Size	No	Yes	No	Yes	Yes
Income steps 2 to 10	No	No	No	No	Yes
Country Effect	No	No	Yes	No	No
N	29104	24331	29104	21325	18396

Ordered Probit Estimation. Data refer to waves 1994-99, 1999-04, 2005-08. Dummy of the last quantile (the 10th) is omitted. Reg.GDP is the per capita regional GDP in PPP, in 10K, 2005 USD. Standard errors are clustered at the quantile level (in brackets).

Table 4. Regional GDP and life satisfaction in EU14 regions

	EU14 1996-2006 b/se	EU14 1996-2006 b/se	EU14 1996-2006 b/se	EU14 1996-2006 b/se	EU14 1996-2006 b/se
1st quantile	–0.2907***	–0.3324***	–0.1042***	–0.1232***	–0.1750***
	(0.0044)	(0.0240)	(0.0272)	(0.0328)	(0.0513)
2nd quantile	–0.0017	–0.0597***	–0.0330	–0.0552*	–0.0950***
	(0.0011)	(0.0192)	(0.0231)	(0.0284)	(0.0291)
3rd quantile	–0.1692***	–0.2787***	–0.0852***	–0.1935***	–0.1909***
	(0.0028)	(0.0255)	(0.0150)	(0.0336)	(0.0435)
4th quantile	–0.0930***	–0.1388***	–0.0136	–0.1327***	–0.1065***
	(0.0015)	(0.0224)	(0.0132)	(0.0312)	(0.0348)
5th quantile	–0.1042***	–0.1090***	–0.0332*	–0.0754***	–0.0784***
	(0.0016)	(0.0148)	(0.0184)	(0.0160)	(0.0244)
6th quantile	–0.0679***	–0.0614***	0.0495*	–0.0638***	–0.0794***
	(0.0010)	(0.0093)	(0.0298)	(0.0098)	(0.0177)
7th quantile	0.0704***	0.0567***	0.0384***	0.0958***	0.0615**
	(0.0007)	(0.0161)	(0.0135)	(0.0192)	(0.0251)
8th quantile	0.0177***	0.0259*	0.0566***	0.0694***	0.0993***
	(0.0007)	(0.0156)	(0.0217)	(0.0213)	(0.0232)
9th quantile	–0.0300***	–0.0070	0.0229	0.0168	–0.0423
	(0.0014)	(0.0140)	(0.0167)	(0.0202)	(0.0264)
Age				–0.0080***	–0.0175***
				(0.0029)	(0.0030)
Age^2				0.0001***	0.0002***
				(0.0000)	(0.0000)
Male				–0.0182	–0.0220
				(0.0239)	(0.0260)
Education	No	No	No	Yes	No
Employment Status	No	No	No	Yes	No
Year Effect	No	No	No	Yes	Yes
Town Size	No	Yes	No	Yes	Yes
Income steps 2 to 10	No	No	No	No	Yes
Country Effect	No	No	Yes	No	No
N	32091	26781	32091	23623	20401

Ordered Probit Estimation. Data refer to waves 1994-99, 1999-04, 2005-08. Dummy of the last quantile (the 10th) is omitted. Reg.GDP is the per capita regional GDP in PPP, in 10K, 2005 USD. Standard errors are clustered at the quantile level (in brackets).

Figure 1. Effect of GDP quantiles on life satisfaction in the 30-quantile partition of all WVS data. Coefficients on the dummies indicate the different 30 quantiles – with the 95% confidence intervals and errors clustered at country and wave levels– derived from the basic ordered probit regression. The Base level is the 30th quantiles. GDP is in 10K, 2005 USD, PPP adjusted

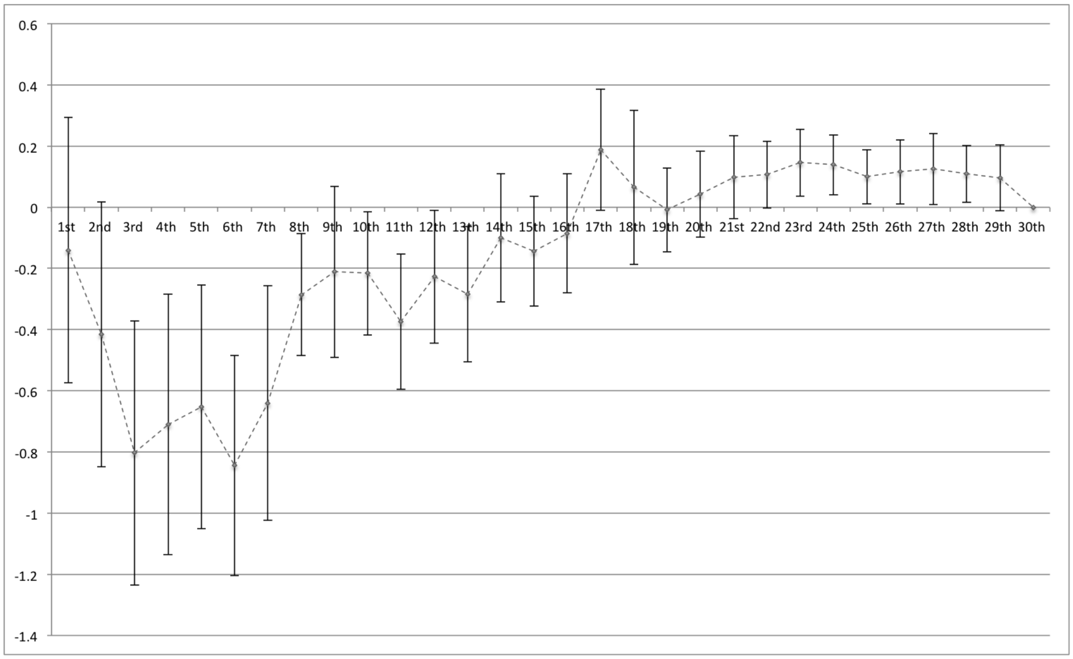

Figure 2. Effect of GDP quantiles on life satisfaction in the 50-quantile partition of all WVS data. Coefficients of the dummies indicate the different 50 quantiles derived from the basic ordered probit regression (controlling for the country specific effect). The continuous line is the estimated cubic interpolation:
$Coefficient = -.51 - 0.023 Quantile + 0.002 Quantile^2 - 0.00003 Quantile^3$ with
$se = [.105; .017; .0008; .00001]$. GDP is in 10K, 2005 USD, PPP adjusted

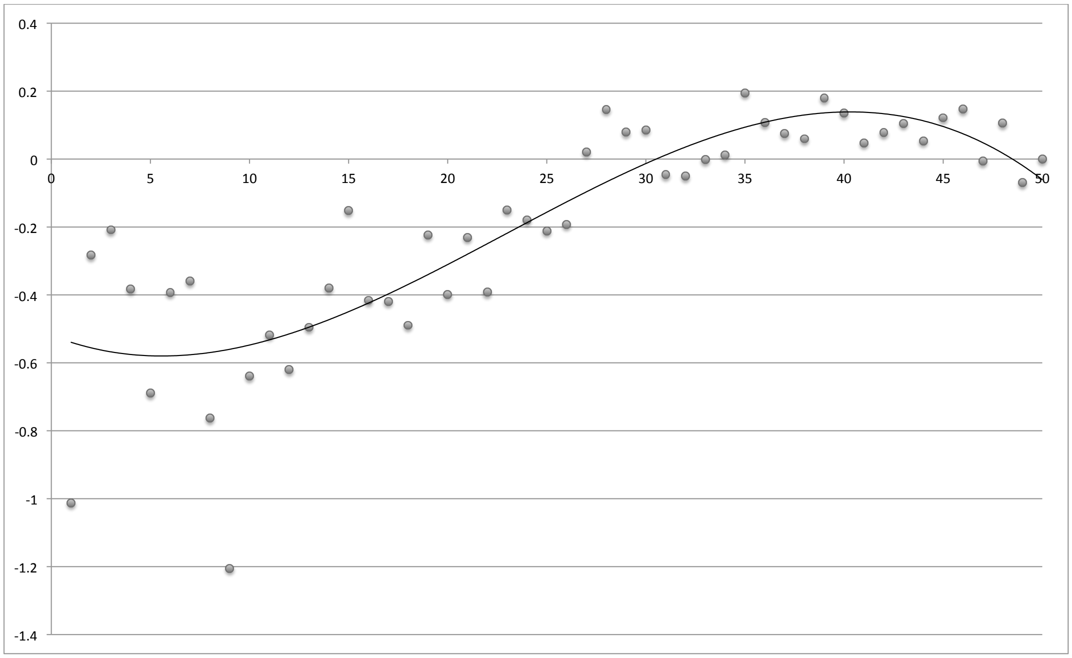

Figure 3. Effect of GDP on life satisfaction the countries above the 25th quantile, in the 50-quantile partition. All Data are partitioned into 50 quantiles, ordered by per capita GDP levels. Each circle represents the ordered probit coefficients of the dummies related to the last 25 quantiles on life satisfaction, controlling for the country specific effect. The continuous line represents a quadratic interpolation with the 95% confidence interval.

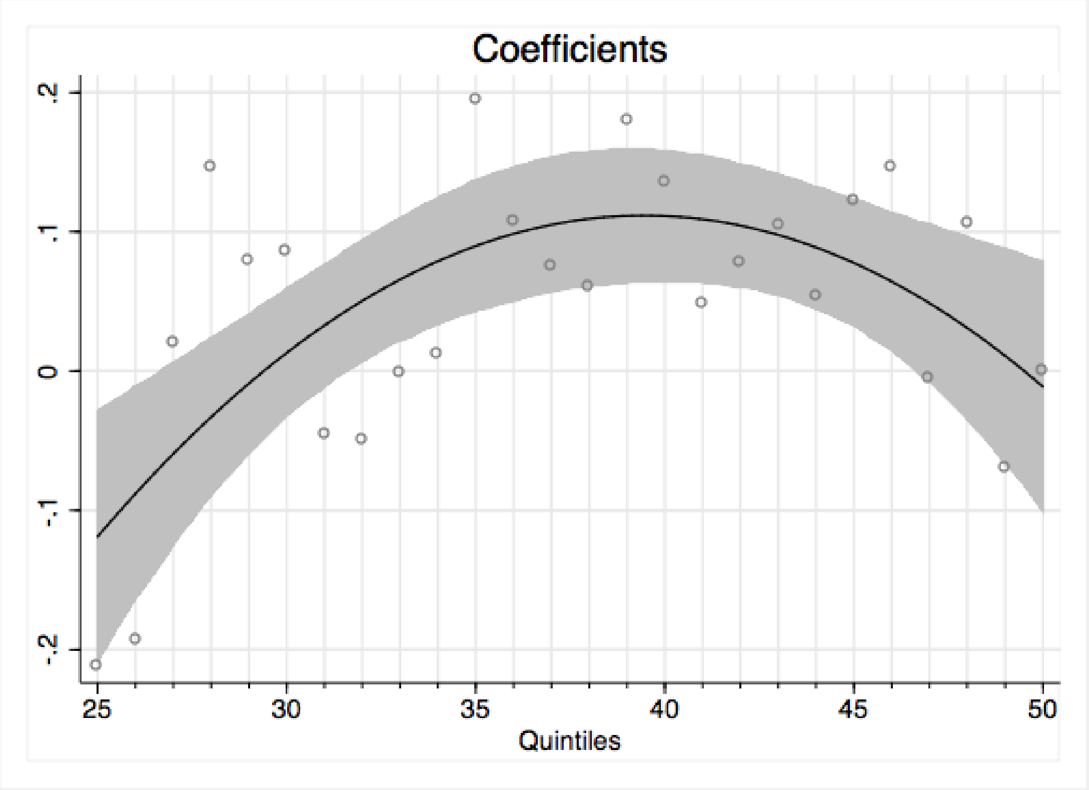

Figure 4. Effect of regional GDP quantiles on life satisfaction in the 10-quantile partition of EU14 data. Coefficients on the dummies indicate the different 10 quantiles–with the 95% confidence intervals and errors clustered at quantile level – derived from the basic ordered probit regression. GDP is in 10K, 2005 USD, PPP adjusted.

A: All data: The base level is the 10th quantile.

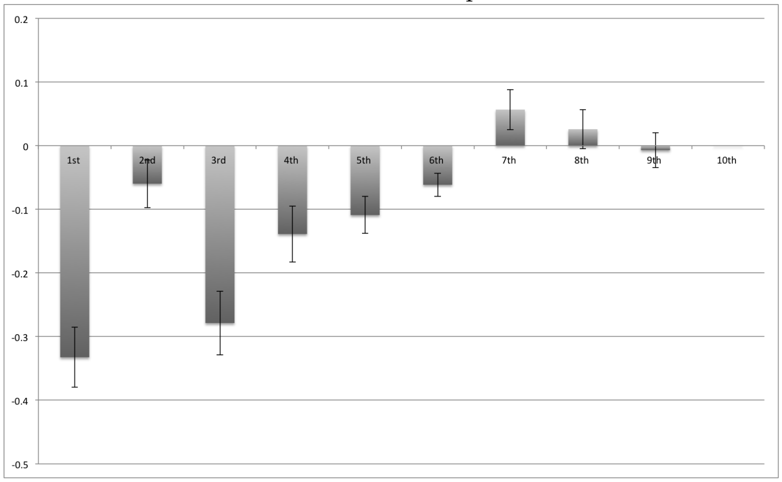

B: Without 10th quantile: The base level is the 9th quantile.

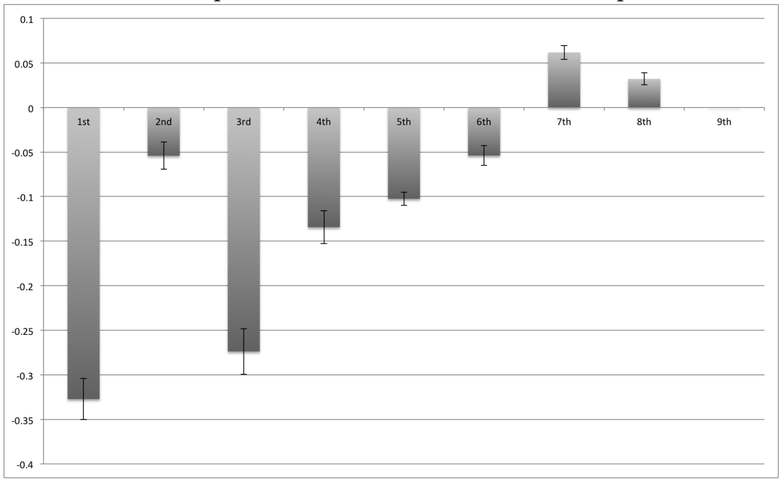

S2: Data Description

Table 5. WVS dataset waves 1982-2006 All Countries, Main Variables.

Variable	Mean	Std. Dev.	Min.	Max.	N
Life Satisfaction	6.645	2.44	1	10	321152
GDP	1.675	1.239	0.027	6.468	311921
Income Steps	4.66	2.442	1	11	282271
Age	41.835	16.385	18	101	316969
Male	0.478	0.499	0	1	321112
Academic achievement	6.53	3.034	1	10	246012

Table 6. WVS dataset waves 1990-2006 EU14, Main Variables

Variable	Mean	Std. Dev.	Min.	Max.	N
Life Satisfaction	7.37	1.934	1	10	56307
Reg.GDP	3.056	1.064	1.471	8.446	32275
Income Steps	4.848	2.555	1	10	44527
Age	44.958	17.34	18	98	56493
Male	0.471	0.499	0	1	56629
size of town	4.895	2.318	1	9	49197
Academic achievement	6.221	2.978	1	10	51946

Table 7. WVS dataset waves 1982-2006 EU14, Derived Variables

Variable	Mean	Std. Dev.	Min.	Max.	N
Log Personal Income	0.367	0.940	-2.774	3.192	32339
Personal Income	2.129	1.948	0.062	24.341	32339
Neuroticism	0	0.792	-1.165	2.639	19694
Extraversion	0	0.861	-2.184	2.312	19694

S3: Countries' 15 Quantiles Partition

Table 8. Country/Wave in the 1st Quantile

	wave			
	1994-1999	1999-2004	2005-2007	Total
Bangladesh	0	1,488	0	1,488
Burkina Faso	0	0	1,470	1,470
Ethiopia	0	0	1,434	1,434
Ghana	0	0	1,477	1,477
India	2,040	0	0	2,040
Kyrgyz Republic	0	1,043	0	1,043
Mali	0	0	1,487	1,487
Moldova	984	0	0	984
Nigeria	1,996	2,022	0	4,018
Rwanda	0	0	1,441	1,441
Tanzania	0	1,157	0	1,157
Uganda	0	1,002	0	1,002
Zambia	0	0	1,377	1,377
Zimbabwe	0	1,002	0	1,002
Total	5,020	7,714	8,686	21,420

Table 9. Country/Wave in the 2nd Quantile

	wave			
	1994-1999	1999-2004	2005-2007	Total
Armenia	1,831	0	0	1,831
Azerbaijan	1,944	0	0	1,944
China	1,500	0	0	1,500
Georgia	1,924	0	0	1,924
India	0	2,002	2,001	4,003
Indonesia	0	996	0	996
Moldova	0	1,008	1,046	2,054
Pakistan	0	2,000	0	2,000
Philippines	1,200	1,200	0	2,400
Vietnam	0	1,000	1,495	2,495
Total	8,399	8,206	4,542	21,147

Table 10. Country/Wave in the 3rd Quantile

	wave			
	1994-1999	1999-2004	2005-2007	Total
Albania	996	1,000	0	1,996
Belarus	2,092	0	0	2,092
Bosnia and Herzegovina	1,200	1,200	0	2,400
China	0	1,000	0	1,000
Georgia	0	0	1,500	1,500
Guatemala	0	0	1,000	1,000
Indonesia	0	0	1,961	1,961
Jordan	0	1,223	1,200	2,423
Morocco	0	2,264	1,200	3,464
Ukraine	2,811	1,195	0	4,006
Total	7,099	7,882	6,861	21,842

Table 11. Country/Wave in the 4th Quantile

	wave					
	1981-1984	1989-1993	1994-1999	1999-2004	2005-2007	Total
Algeria	0	0	0	1,282	0	1,282
Belarus	0	0	0	1,000	0	1,000
Bulgaria	0	0	1,042	999	0	2,041
China	0	0	0	0	2,015	2,015
Egypt, Arab Rep.	0	0	0	0	3,051	3,051
El Salvador	0	0	1,254	0	0	1,254
Korea, Rep.	970	0	0	0	0	970
Latvia	0	0	1,200	0	0	1,200
Macedonia, FYR	0	0	995	0	0	995
Peru	0	0	1,211	1,501	0	2,712
Romania	0	1,103	1,239	1,146	0	3,488
Ukraine	0	0	0	0	1,000	1,000
Total	970	1,103	6,941	5,928	6,066	21,008

Table 12. Country/Wave in the 5th Quantile

	wave				
	1989-1993	1994-1999	1999-2004	2005-2007	Total
Belarus	1,015	0	0	0	1,015
Brazil	1,782	0	0	0	1,782
Bulgaria	1,034	0	0	0	1,034
Colombia	0	6,003	0	3,025	9,028
Latvia	0	0	1,013	0	1,013
Macedonia, FYR	0	0	1,055	0	1,055
Montenegro	0	0	1,060	0	1,060
Serbia	0	1,279	1,200	0	2,479
South Africa	0	2,785	0	0	2,785
Thailand	0	0	0	1,533	1,533
Total	3,831	10,067	4,328	4,558	22,784

Table 13. Country/Wave in the 6th Quantile

	wave					
	1981-1984	1989-1993	1994-1999	1999-2004	2005-2007	Total
Brazil	0	0	1,149	0	0	1,149
Estonia	0	0	1,021	0	0	1,021
Iran, Islamic Rep.	0	0	0	2,406	0	2,406
Lithuania	0	0	1,009	1,018	0	2,027
Peru	0	0	0	0	1,500	1,500
Poland	0	982	0	0	0	982
Russian Federation	0	0	2,040	2,500	0	4,540
South Africa	1,596	0	0	2,828	0	4,424
Turkey	0	1,030	0	4,607	0	5,637
Total	1,596	2,012	5,219	13,359	1,500	23,686

Table 14. Country/Wave in the 7th Quantile

	wave					
	1981-1984	1989-1993	1994-1999	1999-2004	2005-2007	Total
Brazil	0	0	0	0	1,500	1,500
Bulgaria	0	0	0	0	1,001	1,001
Estonia	0	0	0	1,005	0	1,005
Latvia	0	894	0	0	0	894
Malta	467	0	0	0	0	467
Romania	0	0	0	0	1,776	1,776
Serbia	0	0	0	0	1,220	1,220
South Africa	0	0	0	0	2,821	2,821
Turkey	0	0	1,881	0	0	1,881
Uruguay	0	0	1,000	0	1,000	2,000
Venezuela, RB	0	0	1,200	1,200	0	2,400
Total	467	894	4,081	2,205	9,318	16,965

Table 15. Country/Wave in the 8th Quantile

	wave					
	1981-1984	1989-1993	1994-1999	1999-2004	2005-2007	Total
Argentina	0	0	0	1,280	1,002	2,282
Chile	0	0	0	1,200	0	1,200
Croatia	0	0	1,196	1,003	0	2,199
Estonia	0	966	0	0	0	966
Hungary	1,464	999	0	0	0	2,463
Iran, Islamic Rep.	0	0	0	0	2,656	2,656
Korea, Rep.	0	1,251	0	0	0	1,251
Lithuania	0	956	0	0	0	956
Mexico	1,837	0	2,313	0	0	4,150
Poland	0	0	1,153	1,095	0	2,248
Slovak Republic	0	1,135	0	0	0	1,135
Total	3,301	5,307	4,662	4,578	3,658	21,506

Table 16. Country/Wave in the 9th Quantile

	wave					
	1981-1984	1989-1993	1994-1999	1999-2004	2005-2007	Total
Chile	0	0	0	0	1,000	1,000
Czech Republic	0	2,109	0	0	0	2,109
Hungary	0	0	650	1,000	0	1,650
Ireland	1,217	0	0	0	0	1,217
Malaysia	0	0	0	0	1,068	1,068
Malta	0	383	0	0	0	383
Mexico	0	0	0	1,535	1,560	3,095
Poland	0	0	0	0	1,000	1,000
Russian Federation	0	1,961	0	0	1,935	3,896
Slovak Republic	0	466	1,095	1,331	0	2,892
Slovenia	0	1,017	0	0	0	1,017
Spain	2,302	0	0	0	0	2,302
Turkey	0	0	0	0	1,346	1,346
Total	3,519	5,936	1,745	3,866	7,909	22,975

Table 17. Country/Wave in the 10th Quantile

	wave				
	1981-1984	1989-1993	1994-1999	1999-2004	Total
Czech Republic	0	924	1,147	1,907	3,978
Finland	1,003	0	0	0	1,003
Ireland	0	1,000	0	0	1,000
Italy	1,345	0	0	0	1,345
Japan	1,204	0	0	0	1,204
Korea, Rep.	0	0	1,247	1,200	2,447
Malta	0	0	0	1,000	1,000
Portugal	0	1,185	0	1,000	2,185
Slovenia	0	0	1,007	1,006	2,013
Spain	0	4,147	0	0	4,147
United Kingdom	1,167	0	0	0	1,167
Total	4,719	7,256	3,401	6,113	21,489

Table 18. Country/Wave in the 11th Quantile

	wave				
	1981-1984	1994-1999	1999-2004	2005-2007	Total
Australia	1,157	0	0	0	1,157
Belgium	1,138	0	0	0	1,138
Canada	1,254	0	0	0	1,254
Denmark	1,182	0	0	0	1,182
Finland	0	981	0	0	981
France	1,198	0	0	0	1,198
Germany	1,303	0	0	0	1,303
Greece	0	0	1,142	0	1,142
Iceland	927	0	0	0	927
Israel	0	0	1,199	0	1,199
Korea, Rep.	0	0	0	1,200	1,200
Netherlands	1,221	0	0	0	1,221
New Zealand	0	1,201	0	0	1,201
Saudi Arabia	0	0	1,427	0	1,427
Spain	0	1,211	0	0	1,211
Sweden	954	0	0	0	954
Trinidad and Tobago	0	0	0	1,002	1,002
Total	10,334	3,393	3,768	2,202	19,697

Table 19. Country/Wave in the 12th Quantile

	wave					
	1981-1984	1989-1993	1994-1999	1999-2004	2005-2007	Total
Austria	0	1,460	0	0	0	1,460
Belgium	0	2,790	0	0	0	2,790
Cyprus	0	0	0	0	1,049	1,049
Denmark	0	1,030	0	0	0	1,030
Finland	0	588	0	0	0	588
France	0	1,002	0	0	0	1,002
Iceland	0	702	0	0	0	702
Italy	0	2,018	0	0	0	2,018
New Zealand	0	0	0	0	954	954
Slovenia	0	0	0	0	1,037	1,037
Spain	0	0	0	2,409	0	2,409
Sweden	0	1,047	1,009	0	0	2,056
United Kingdom	0	1,484	0	0	0	1,484
United States	2,325	0	0	0	0	2,325
Total	2,325	12,121	1,009	2,409	3,040	20,904

Table 20. Country/Wave in the 13th Quantile

	wave					
	1981-1984	1989-1993	1994-1999	1999-2004	2005-2007	Total
Australia	0	0	1,945	0	0	1,945
Canada	0	1,730	0	0	0	1,730
Finland	0	0	0	1,036	0	1,036
France	0	0	0	1,615	0	1,615
Germany	0	3,437	2,026	0	0	5,463
Italy	0	0	0	2,000	1,012	3,012
Japan	0	1,011	1,054	0	0	2,065
Netherlands	0	1,017	0	0	0	1,017
Norway	1,031	0	0	0	0	1,031
Sweden	0	0	0	1,015	0	1,015
United Kingdom	0	0	1,051	0	0	1,051
Total	1,031	7,195	6,076	5,666	1,012	20,980

Table 21. Country/Wave in the 14th Quantile

	wave				
	1989-1993	1994-1999	1999-2004	2005-2007	Total
Austria	0	0	1,522	0	1,522
Belgium	0	0	1,906	0	1,906
Denmark	0	0	1,023	0	1,023
Finland	0	0	0	1,013	1,013
France	0	0	0	1,001	1,001
Germany	0	0	2,036	2,064	4,100
Iceland	0	0	968	0	968
Ireland	0	0	1,012	0	1,012
Japan	0	0	1,362	1,096	2,458
Norway	1,239	0	0	0	1,239
Spain	0	0	0	1,200	1,200
Switzerland	0	1,212	0	0	1,212
United Kingdom	0	0	998	0	998
United States	1,838	0	0	0	1,838
Total	3,077	1,212	10,827	6,374	21,490

Table 22. Country/Wave in the 15th Quantile

	wave				
	1989-1993	1994-1999	1999-2004	2005-2007	Total
Australia	0	0	0	1,421	1,421
Canada	0	0	1,931	2,148	4,079
Luxembourg	0	0	1,161	0	1,161
Netherlands	0	0	1,003	1,002	2,005
Norway	0	1,127	0	1,025	2,152
Singapore	0	0	1,300	0	1,300
Sweden	0	0	0	1,003	1,003
Switzerland	1,400	0	0	1,241	2,641
United Kingdom	0	0	0	1,011	1,011
United States	0	1,542	1,200	1,249	3,991
Total	1,400	2,669	6,595	10,100	20,764

S4: Regions' 5 Quantiles Partition

Table 23. Region/Wave in the 1st Quantile

	wave			
	1994-1999	1999-2004	2005-2007	Total
Aitoloakarnania	0	20	0	20
Alentejo	0	55	0	55
Andalucia	214	432	0	646
Argolida	0	5	0	5
Asturias	34	0	0	34
Brandenburg	173	170	0	343
Calabria	0	66	25	91
Campania	0	185	102	287
Cantabria	51	0	0	51
Castilla-la Mancha	77	103	0	180
Centro (PT)	0	185	0	185
Chios	0	10	0	10
Etel-Karjala	58	0	0	58
Etel-Pohjanmaa	0	54	0	54
Etel-Savo	35	33	0	68
Evvoia	0	36	0	36
Extremadura	33	64	27	124
Galicia	86	170	0	256

Table 24. Region/Wave in the 1st Quantile cont'd

	wave			
	1994-1999	1999-2004	2005-2007	Total
Kainuu	0	21	0	21
Kanta-Hme	141	0	0	141
Karditsa	0	26	0	26
Kerkyra	0	15	0	15
Keski-Suomi	48	0	0	48
Lakonia	0	12	0	12
Lappi	49	0	0	49
Larisa	0	5	0	5
Luxembourg (Grand-Duch)	0	1,161	0	1,161
Magnisia	0	27	0	27
Messinia	0	2	0	2
Murcia	33	0	0	33
Norte	0	355	0	355
Northern Ireland	64	0	0	64
Pohjois-Karjala	36	45	0	81
Pohjois-Pohjanmaa	100	0	0	100
Pohjois-Savo	54	0	0	54
Puglia	0	136	71	207
Sachsen	296	290	0	586
Sachsen-Anhalt	169	175	0	344
Sicilia	0	168	88	256
Thessalia	0	1	0	1
Thringen	162	155	0	317
Trikala	0	40	0	40
Wales	43	0	0	43
Total	1,956	4,222	313	6,491

Table 25. Region/Wave in the 2nd Quantile

	wave			
	1994-1999	1999-2004	2005-2007	Total
Abruzzo	0	0	16	16
Algarve	0	40	0	40
Andalucia	0	0	209	209
Arkadia	0	4	0	4
Asturias	0	68	33	101
Basilicata	0	21	11	32
Berlin	123	0	0	123
Brandenburg	0	0	200	200
Burgenland (A)	0	65	0	65
Canarias (ES)	191	97	51	339
Cantabria	0	32	0	32
Castilla y Len	16	151	0	167
Castilla-la Mancha	0	0	49	49
Chania	0	7	0	7
Comunidad Valenciana	122	244	124	490
East Midlands (ENGLAND)	74	0	0	74
Eastern	40	0	0	40
Etel-Pohjanmaa	0	0	64	64
Etel-Savo	0	0	36	36
Flevoland	0	17	0	17
Fthiotida	0	5	0	5
Galicia	0	0	84	84
Kainuu	0	0	11	11
Kanta-Hme	0	68	40	108
Keski-Pohjanmaa	0	11	0	11
Keski-Suomi	0	62	0	62
Kriti	0	15	0	15
La Rioja	8	0	0	8
Lappi	0	22	0	22
Molise	0	29	13	42
Murcia	0	66	35	101
Mditerrane	0	235	0	235
Niedersachsen	104	0	0	104
Nord	0	84	0	84
North East (ENGLAND)	0	56	0	56
North West (ENGLAND)	108	0	0	108

Table 26. Region/Wave in the 2nd Quantile Cont'd

	wave			
Ouest	0	201	0	201
Pohjanmaa	55	0	0	55
Pohjois-Karjala	0	0	32	32
Pohjois-Pohjanmaa	0	79	0	79
Pohjois-Savo	0	55	58	113
Prov. Hainaut	0	265	0	265
Prov. Lige	0	177	0	177
Prov. Luxembourg (B)	0	44	0	44
Prov. Namur	0	61	0	61
Pijt-Hme	0	33	37	70
Rheinland-Pfalz	60	0	0	60
Saarland	18	0	0	18
Sachsen	0	0	315	315
Sachsen-Anhalt	0	0	176	176
Sardegna	0	57	30	87
Satakunta	0	61	0	61
Schleswig-Holstein	41	0	0	41
Scotland	111	0	0	111
Sjlland	0	164	0	164
South East	203	0	0	203
South West (ENGLAND)	100	0	0	100
Sud-Ouest	0	163	0	163
Thringen	0	0	179	179
Varsinais-Suomi	174	0	0	174
Wales	0	59	50	109
West Midlands (ENGLAND)	105	0	0	105
Yorkshire and The Humber	92	0	0	92
Zaragoza	37	0	0	37
Total	1,782	2,818	1,853	6,453

Table 27. Region/Wave in the 3rd Quantile

	wave			
	1994-1999	1999-2004	2005-2007	Total
Abruzzo	0	29	0	29
Attiki	0	868	0	868
Baden-Wrttemberg	160	0	0	160
Bassin Parisien	0	324	0	324
Bayern	173	0	0	173
Berlin	0	135	101	236
Cantabria	0	0	16	16
Castilla y Len	0	0	75	75
Catalua	46	0	0	46
Centre-Est	0	209	0	209
Dodekanisos	0	17	0	17
Drenthe	0	31	29	60
East Midlands (ENGLAND)	0	61	76	137
Eastern	0	45	0	45
Est	0	100	88	188
Etel-Karjala	0	29	16	45
Flevoland	0	0	17	17
Friesland (NL)	0	32	40	72
Gvleborgs ln	0	0	25	25
Hallands ln	0	0	33	33
Illes Balears	23	0	0	23
Kalmar ln	0	0	17	17
Keski-Suomi	0	0	56	56
Kyklades	0	4	0	4
Kymenlaakso	0	38	0	38
Krnten	0	108	0	108
Lappi	0	0	40	40
Mditerrane	0	0	121	121
Navarra	16	0	0	16
Niedersachsen	0	126	137	263
Niedersterreich	0	315	0	315
Nord	0	0	68	68
Nordrhein-Westfalen	293	0	0	293
North West (ENGLAND)	0	138	68	206

Table 28. Region/Wave in the 3rd Quantile cont'd

	**wave**			
	1994-1999	1999-2004	2005-2007	Total
Northern Ireland	0	0	90	90
Ouest	0	0	136	136
Pais Vasco	64	0	0	64
Pirkanmaa	0	67	0	67
Pohjanmaa	0	10	0	10
Pohjois-Pohjanmaa	0	0	64	64
Prov. Limburg (B)	0	101	0	101
Prov. Oost-Vlaanderen	0	208	0	208
Rheinland-Pfalz	0	54	70	124
Saarland	0	16	0	16
Satakunta	0	0	56	56
Schleswig-Holstein	0	23	40	63
Scotland	0	83	0	83
South West (ENGLAND)	0	79	0	79
Steiermark	0	221	0	221
Sud-Ouest	0	0	109	109
Sdermanlands ln	0	0	24	24
Umbria	0	0	15	15
Varsinais-Suomi	0	97	0	97
Vrmlands ln	0	0	12	12
Vstmanlands ln	0	0	5	5
West Midlands (ENGLAND)	0	99	110	209
Yorkshire and The Humber	0	60	84	144
Zaragoza	0	74	0	74
stergtlands ln	0	0	7	7
Total	775	3,801	1,845	6,421

Table 29. Region/Wave in the 4th Quantile

	wave			
	1994-1999	1999-2004	2005-2007	Total
Baden-Wrttemberg	0	160	0	160
Bayern	0	181	0	181
Catalua	0	376	192	568
Dalarnas ln	0	0	19	19
Eastern	0	0	43	43
Friuli-Venezia Giulia	0	43	22	65
Gelderland	0	145	107	252
Grande Lisboa	0	365	0	365
Hessen	89	0	0	89
Illes Balears	0	47	25	72
Jmtlands ln	0	0	2	2
Jnkpings ln	0	0	77	77
Korinthia	0	19	0	19
Kronobergs ln	0	0	22	22
Kymenlaakso	0	0	38	38
La Rioja	0	16	8	24
Lazio	0	0	97	97
Liguria	0	61	28	89
Limburg (NL)	0	34	74	108
Madrid	160	0	0	160
Marche	0	47	17	64
Midtjylland	0	235	0	235
Navarra	0	33	15	48
Nordjylland	0	109	0	109
Nordrhein-Westfalen	0	289	252	541
Norrbottens ln	0	0	11	11
Obersterreich	0	271	0	271
Overijssel	0	76	71	147
Pais Vasco	0	130	0	130
Piemonte	0	147	70	217
Pirkanmaa	0	0	103	103
Pohjanmaa	0	0	16	16
Prov. Brabant Wallon	0	45	0	45
Prov. Vlaams Brabant	0	104	0	104
Prov. West-Vlaanderen	0	164	0	164

Table 30. Region/Wave in the 4th Quantile cont'd

	wave			
	1994-1999	1999-2004	2005-2007	Total
Saarland	0	0	9	9
Scotland	0	0	113	113
Skne ln	0	0	135	135
South East	0	187	0	187
South West (ENGLAND)	0	0	108	108
Syddanmark	0	243	0	243
Tirol	0	80	0	80
Toscana	0	133	65	198
Umbria	0	30	0	30
Uppsala ln	0	0	57	57
Uusimaa	231	0	0	231
Valle d'Aosta/Valle d'Aoste	0	0	12	12
Varsinais-Suomi	0	0	88	88
Vorarlberg	0	55	0	55
Vsterbottens ln	0	0	40	40
Vsternorrlands ln	0	0	40	40
Vstra Gtalands ln	0	0	217	217
Zaragoza	0	0	32	32
Zeeland	0	23	22	45
rebro ln	0	0	3	3
Total	480	3,848	2,250	6,578

Table 31. Region/Wave in the 5th Quantile

	wave			
	1994-1999	1999-2004	2005-2007	Total
Baden-Wrttemberg	0	0	149	149
Bayern	0	0	185	185
Emilia-Romagna	0	145	74	219
Groningen	0	37	35	72
Hamburg	30	20	23	73
Hessen	0	103	70	173
Hovedstaden	0	272	0	272
Lazio	0	181	0	181
Lombardia	0	320	154	474
London	111	90	68	269
Madrid	0	306	158	464
Noord-Brabant	0	151	149	300
Noord-Holland	0	206	164	370
Pais Vasco	0	0	67	67
Paris	0	0	361	361
Prov. Antwerpen	0	242	0	242
Rgion de Bruxelles-Capitale	0	495	0	495
Salzburg	0	98	0	98
South East	0	0	201	201
Stockholms ln	0	0	257	257
Trentino Alto Adige	0	33	18	51
Utrecht	0	34	69	103
Uusimaa	0	251	258	509
Valle d'Aosta/Valle d'Aoste	0	10	0	10
Voiotia	0	1	0	1
Zuid-Holland	0	212	225	437
le de France	0	299	0	299
Total	141	3,506	2,685	6,332

S5: Factor Analysis to Determine the Personality Traits

Determination of traits from the score in each question was necessary because no existing imputation to traits on the sample of questions in the data exists.

Trait determination was realized with exploratory factor analysis (statistical software Stata, release 11). We retained factors with eigenvalues larger than a threshold value of 1 as suggested by different sources. We selected all the personality questions in the WVS dataset, such questions were available only for the wave 1989-93. For completeness, we also included the variable e065, the answer 'none of the above' to the residual question. In Figure 5 we present the list of the questions and some descriptive statistics. In Figure 6 we present the Stata log showing the eigenvalues of all factors and the factor loadings.

We plot the factors' loadings with eigenvalues larger than 1 in Figure 7, where we note that variables are clustered into two main groups. A high score in the group of variable with high loading on factor 1 represents high excitement and assertiveness, high seeking of stimulation and other peoples' company, and a pronounced engagement with the external world. We therefore defined factor 1 as Extraversion. A high score in the group of variables with high loading on factor 2 represents negative emotions like depression, loneliness, boredom, anxiousness, and anger. We defined factor 2 as neuroticism.

To complete the analysis we also present the rotated matrix in Figure 8 and the Kaiser-Meyer-Olkin measure of sampling adequacy in Figure 9. This test generates values between 0 and 1 for each variable included, with smaller values meaning the variables have too little in common to warrant a factor analysis. All our variables show adequacy levels well above 0.7, generally considered the acceptable threshold.

Figure 5. Questions used for the determination of personality traits

Variable	Obs	Unique	Mean	Min	Max	Label
a010	21116	2	.5107028	0	1	ever felt very excited or interested
a011	21103	2	.3086765	0	1	ever felt restless
a012	21093	2	.463092	0	1	ever felt proud because someone complimented you
a013	21076	2	.1897419	0	1	ever felt very lonely or remote from other people
a014	21074	2	.7216475	0	1	ever felt pleased about having accomplished something
a015	21087	2	.2185233	0	1	ever felt bored
a016	21026	2	.3521355	0	1	ever felt on top of the world
a017	21060	2	.2213675	0	1	ever felt depressed or very unhappy
a018	21017	2	.4764238	0	1	ever felt that things were going your way
a019	21056	2	.1745821	0	1	ever felt upset because somebody criticized you
e047	20404	10	.5806558	.1	1	personal characteristics: changes, worry or welcome possibility
e048	21113	2	.4707053	0	1	personal characteristics: i usually count on being successful in everything I do
e049	21134	2	.2725466	0	1	personal characteristics: i enjoy convincing others of my opinion
e050	21070	2	.1612245	0	1	personal characteristics: i serve as a model for others
e051	21096	2	.3396378	0	1	personal characteristics: i am good at getting what i want
e052	21088	2	.1323976	0	1	personal characteristics: i own many things others envy me for
e053	21146	2	.5175447	0	1	personal characteristics: i like to assume responsibility
e054	21132	2	.3680201	0	1	personal characteristics: i am rarely unsure about how i should behave
e055	21146	2	.4250449	0	1	personal characteristics: i often give others advice
e056	21205	2	.1211035	0	1	personal characteristics: none of the above

Figure 6. Factor Analysis

```
. factor  a010 a011 a012 a013 a014 a015 a016 a017 a018 a019 e047 e048 e049 e050 e051 e052 e053 e054 e055
(obs=19694)

Factor analysis/correlation                        Number of obs    =    19694
    Method: principal factors                      Retained factors =        2
    Rotation: (unrotated)                          Number of params =       39
```

Factor	Eigenvalue	Difference	Proportion	Cumulative
Factor1	2.31880	0.98316	0.7000	0.7000
Factor2	1.33564	0.67889	0.4032	1.1032
Factor3	0.65675	0.43986	0.1983	1.3014
Factor4	0.21689	0.07117	0.0655	1.3669
Factor5	0.14572	0.06469	0.0440	1.4109
Factor6	0.08103	0.03675	0.0245	1.4354
Factor7	0.04428	0.05078	0.0134	1.4487
Factor8	-0.00651	0.01437	-0.0020	1.4468
Factor9	-0.02088	0.01448	-0.0063	1.4405
Factor10	-0.03536	0.02370	-0.0107	1.4298
Factor11	-0.05906	0.02329	-0.0178	1.4120
Factor12	-0.08235	0.01896	-0.0249	1.3871
Factor13	-0.10131	0.01390	-0.0306	1.3565
Factor14	-0.11521	0.01518	-0.0348	1.3217
Factor15	-0.13039	0.01064	-0.0394	1.2824
Factor16	-0.14103	0.01303	-0.0426	1.2398
Factor17	-0.15406	0.02394	-0.0465	1.1933
Factor18	-0.17800	0.03131	-0.0537	1.1396
Factor19	-0.20931	0.04372	-0.0632	1.0764
Factor20	-0.25303	.	-0.0764	1.0000

```
    LR test: independent vs. saturated: chi2(190) = 4.4e+04 Prob>chi2 = 0.0000

Factor loadings (pattern matrix) and unique variances
```

Variable	Factor1	Factor2	Uniqueness
a010	0.4021	0.1101	0.8262
a011	0.2037	0.4096	0.7908
a012	0.4533	0.1133	0.7817
a013	0.0006	0.5681	0.6772
a014	0.4643	0.0023	0.7844
a015	-0.0663	0.4165	0.8221
a016	0.4051	-0.0188	0.8356
a017	0.0405	0.6166	0.6182
a018	0.3115	-0.2511	0.8399
a019	0.1156	0.3614	0.8560
e047	0.2304	-0.1206	0.9324
e048	0.3785	-0.0945	0.8478
e049	0.3516	0.0685	0.8717
e050	0.3449	0.0484	0.8787
e051	0.4160	-0.0785	0.8208
e052	0.2861	0.0687	0.9134
e053	0.4443	-0.0934	0.7939
e054	0.2830	-0.1099	0.9078
e055	0.3853	0.0545	0.8486
e056	-0.5425	0.0856	0.6984


```
. log close
      name:
       log:
  log type:
 closed on:
```

Figure 7. Factor Loadings of the Personality Factor Analysis. Factor 1 has been defined as extraversion, Factor 2 has been defined as neuroticism, variable e065 is the answer 'none of the above' to the residual questions

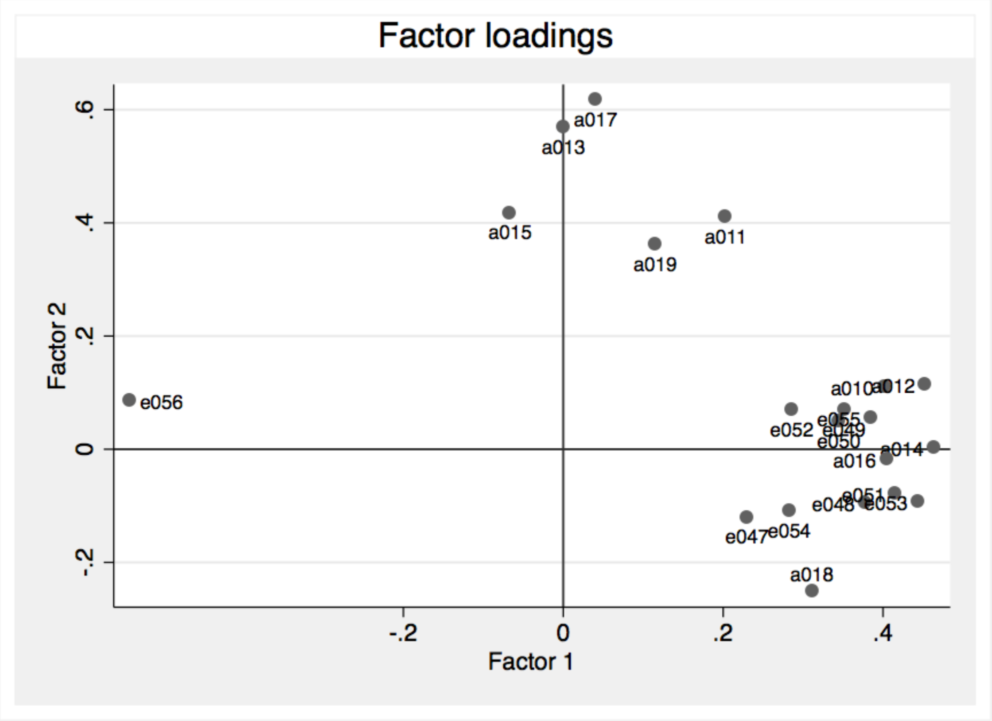

Figure 8. Rotated Matrix of Correlation

```
. rotate

Factor analysis/correlation                        Number of obs    =    19694
    Method: principal factors                      Retained factors =        2
    Rotation: orthogonal varimax (Kaiser off)      Number of params =       39
```

Factor	Variance	Difference	Proportion	Cumulative
Factor1	**2.31470**	**0.97495**	**0.6987**	**0.6987**
Factor2	**1.33975**	**.**	**0.4044**	**1.1032**

LR test: independent vs. saturated: chi2(**190**) = **4.4e+04** Prob>chi2 = **0.0000**

Rotated factor loadings (pattern matrix) and unique variances

Variable	Factor1	Factor2	Uniqueness
a010	**0.3942**	**0.1359**	**0.8262**
a011	**0.1768**	**0.4219**	**0.7908**
a012	**0.4450**	**0.1423**	**0.7817**
a013	**-0.0362**	**0.5670**	**0.6772**
a014	**0.4632**	**0.0323**	**0.7844**
a015	**-0.0931**	**0.4113**	**0.8221**
a016	**0.4054**	**0.0075**	**0.8356**
a017	**0.0006**	**0.6179**	**0.6182**
a018	**0.3271**	**-0.2304**	**0.8399**
a019	**0.0920**	**0.3681**	**0.8560**
e047	**0.2377**	**-0.1054**	**0.9324**
e048	**0.3838**	**-0.0699**	**0.8478**
e049	**0.3465**	**0.0911**	**0.8717**
e050	**0.3411**	**0.0706**	**0.8787**
e051	**0.4202**	**-0.0515**	**0.8208**
e052	**0.2810**	**0.0870**	**0.9134**
e053	**0.4494**	**-0.0645**	**0.7939**
e054	**0.2895**	**-0.0913**	**0.9078**
e055	**0.3810**	**0.0793**	**0.8486**
e056	**-0.5469**	**0.0503**	**0.6984**

Factor rotation matrix

	Factor1	Factor2
Factor1	**0.9979**	**0.0646**
Factor2	**-0.0646**	**0.9979**

Figure 9. Kaiser-Meyer-Olkin measure of sampling adequacy.

```
. estat kmo

Kaiser-Meyer-Olkin measure of sampling adequacy
```

Variable	kmo
a010	**0.8417**
a011	**0.8015**
a012	**0.8102**
a013	**0.7172**
a014	**0.8197**
a015	**0.7763**
a016	**0.8030**
a017	**0.7057**
a018	**0.7816**
a019	**0.8085**
e047	**0.8338**
e048	**0.7807**
e049	**0.8639**
e050	**0.8152**
e051	**0.8599**
e052	**0.8325**
e053	**0.8231**
e054	**0.8131**
e055	**0.8053**
e056	**0.7599**
Overall	**0.7963**
